# Sequestosome-1 (SQSTM1/p62) as a target in dopamine catabolite-mediated cellular dyshomeostasis

**DOI:** 10.1038/s41419-024-06763-x

**Published:** 2024-06-18

**Authors:** Anna Masato, Annapaola Andolfo, Giulia Favetta, Edoardo Niccolò Bellini, Susanna Cogo, Luisa Dalla Valle, Daniela Boassa, Elisa Greggio, Nicoletta Plotegher, Luigi Bubacco

**Affiliations:** 1 Department of Biology, University of Padova, Padova, Italy; 2 Proteomics and Metabolomics Facility (ProMeFa), Center for Omics Sciences (COSR), IRCCS San Raffaele Scientific Institute, Milan, Italy; 3 Department of Neurosciences and National Center for Microscopy and Imaging Research, University of California San Diego, La Jolla, CA USA; 4 Centro Studi per la Neurodegenerazione (CESNE), University of Padova, Padova, Italy; 5 Present Address: UK Dementia Research Institute at University College London, London, UK; 6 Present Address: School of Biological Sciences, University of Reading, Reading, UK

**Keywords:** Molecular neuroscience, Neurochemistry

## Abstract

Alterations in the dopamine catabolic pathway are known to contribute to the degeneration of nigrostriatal neurons in Parkinson’s disease (PD). The progressive cellular buildup of the highly reactive intermediate 3,4-dihydroxyphenylacetaldehye (DOPAL) generates protein cross-linking, oligomerization of the PD-linked αSynuclein (αSyn) and imbalance in protein quality control. In this scenario, the autophagic cargo sequestome-1 (SQSTM1/p62) emerges as a target of DOPAL-dependent oligomerization and accumulation in cytosolic clusters. Although DOPAL-induced oxidative stress and activation of the Nrf2 pathway promote p62 expression, p62 oligomerization rather seems to be a consequence of direct DOPAL modification. DOPAL-induced p62 clusters are positive for ubiquitin and accumulate within lysosomal-related structures, likely affecting the autophagy-lysosomal functionality. Finally, p62 oligomerization and clustering is synergistically augmented by DOPAL-induced αSyn buildup. Hence, the substantial impact on p62 proteostasis caused by DOPAL appears of relevance for dopaminergic neurodegeneration, in which the progressive failure of degradative pathways and the deposition of proteins like αSyn, ubiquitin and p62 in inclusion bodies represent a major trait of PD pathology.

## Introduction

Parkinson’s disease (PD) is a severe age-related neurodegenerative disorder, currently affecting more than 10 million people worldwide (source: https://www.parkinson.org) [1]. It is associated with typical motor symptoms that patients display at the time of clinical diagnosis, such as tremor at rest, rigidity, akinesia, and postural instability [2]. This is caused by the progressive loss of the nigrostriatal neurons in the central nervous system with more than 80% decrease in dopamine release within the striatum [3]. This in turn affects the neuronal transmissions of the basal ganglia which are responsible for the control of voluntary movement. At the histopathological level, misfolded and aggregated proteins accumulate along the neurites and in the cell body of neurons into insoluble inclusions named Lewy Neurites (LNs) and Lewy Bodies (LBs), with fibrillar αSynuclein (αSyn) as main constituent [4]. The accumulation of the toxic aggregates further triggers a cascade of many neurotoxic downstream effects, above all failure of degradative systems, mitochondrial dysfunction, calcium imbalance and oxidative stress that hinder neuronal homeostasis at multiple levels [5].

The alteration of dopamine metabolic pathway is among the molecular determinants proposed to account for the selective vulnerability of the dopaminergic neurons in PD [6]. Several studies in PD patient brains revealed a decreased expression and activity of the catabolic enzyme aldehyde dehydrogenase 1A1 (ALDH1A1) [7, 8], which is responsible for the degradation of the dopamine intermediate 3,4-dihydroxyphenylacetaldehyde (DOPAL) generated by monoamine oxidase (MAO). Coherently, accumulation of DOPAL has been demonstrated to be highly neurotoxic for dopaminergic neurons [9, 10]. Indeed, DOPAL and dopamine share similar redox property for the catechol group, but DOPAL is by far more reactive toward proteins due to the aldehydic moiety, thus leading to impaired proteostasis, oxidative stress and neuronal death [6]. In this frame, DOPAL has been demonstrated to covalently modify αSyn lysines triggering its aggregation into SDS-resistant annular-shaped *off-pathway* oligomers [11–13]. DOPAL-induced αSyn oligomeric species are more resistant to degradation and their aberrant accumulation at the pre-synaptic sites, along the neurites and in the soma of dopaminergic neurons results in decreased synaptic integrity, reduced axonal arborization and overwhelming protein quality control systems [14]. A synergistic interplay between DOPAL and αSyn leads to the accumulation of ubiquitinated proteins and endo-lysosomal structures. On the other hand, DOPAL buildup was observed to significantly impact on the levels of the autophagic cargo Sequestosome-1 (SQSTM1 or p62, from now on) also in an αSyn-null background as well [14].

p62 is a scaffold protein of 440 amino acids ubiquitously expressed in all tissues and cell types. It is a multidomain protein, consisting at the N-terminus of a Phox 1 and Bem1p (PB1, aa 21–103) domain, which mediates p62 homodimerization, and a ZZ-type zinc finger (ZZ, aa 128–163) domain; an intrinsic unfolded domain containing two nuclear localization signals (NLS), a TRAF6-binding domain (TB) and a nuclear export signal (NES); an LC3-interacting region (LIR, aa 321–345), a Keap1-interacting region (KIR, aa 346–385) and an ubiquitin-associated domain (UBA, aa 386–440) at the C-terminus [15]. p62 is mostly known for its role in selective autophagy, where it binds ubiquitinated proteins or organelles (K63 polyubiquitin chains) to be engulfed by the autophagosomal membrane and be directed to lysosomal degradation. However, p62 is at the crossroads of several intersecting pathways, i.e., antioxidative stress response via Keap1–Nrf2 axis, inflammation via TRAF6 and NF-kB pathway, apoptosis, ERK1-mediated adipogenesis and nutrient sensing [15]. Both p62 functions and regulations are mediated by a plethora of interacting partners and by several post-translational modifications (PTMs) such as phosphorylation, acetylation, ubiquitination, and cysteine oxidation [16, 17], which render p62 a central hub of cellular homeostasis [18].

p62-impaired proteostasis has been associated with many diseases, among which proteinopathies and neurodegenerative disorders. Several missense mutations on p62 sequence have been identified in Paget’s disease of bone (PDB), amyotrophic lateral sclerosis (ALS) and frontotemporal dementia (FTD) [19], correlating with abnormalities in autophagy, NF-kB and Nrf2 signaling pathways [15]. In the context of PD, loss-of-function of p62 contributes to dysregulated proteasome- and autophagy-mediated protein degradation, leading to aberrant accumulation of misfolded proteins and dysfunctional organelles in dopaminergic neurons [15, 20, 21]. Accordingly, a consistent incorporation of p62 together with ubiquitin and αSyn aggregates has been detected in LBs from PD patients [22], an observation that has been further corroborated by experimental models showing p62 participation in the early steps of LBs formation [23].

In this study, we dissected the molecular mechanisms of DOPAL-induced p62 accumulation. Based on the increased levels of p62 observed in in vivo models of impaired DOPAL detoxification, we demonstrated that acute DOPAL cellular exposure causes a dose-dependent p62 oligomerization and cytosolic clustering, which likely depends on a combination of redox signaling and Nrf2 pathway activation, DOPAL direct modification and cross-linking, and impaired protein clearance. We further show that αSyn buildup induced by DOPAL amplifies p62 oligomerization, supporting the relevance of DOPAL neurotoxicity in dopaminergic neurons in the early phases of PD pathology.

## Materials and methods

### Compounds

DOPAL was synthesized from epinephrine and characterized as previously described, according to Fellman’s protocol [14, 24]. Alternatively, DOPAL was purchased at Cayman Chemical Company (#18448) and resuspended in distilled water to a final concentration of 100 mM. Dopamine hydrochloride (H8502), N-acetylcysteine (A7250), Ascorbic acid (A7506), chloroquine (C6628) and dimethyl fumarate (242926), Disulfiram (86720) and doxycycline (D9891) were purchased at Sigma-Aldrich. Glutathione (6832.4), and Hydrogen peroxide (8070.1) were purchased at CarlRoth. G418 was purchased at InvivoGen.

### Antibodies

The following antibodies were used for western blot and immunofluorescence: mouse anti-β-Actin (A1978, Sigma-Aldrich), rabbit anti-p62 (ab109012, Abcam), rabbit anti-p62 (P0069, Sigma-Aldrich), mouse anti-Ubiquitin (P4D1 sc-8017, Santa Cruz Biotech.), mouse anti-HSP70 (H5147, Sigma-Aldrich), rabbit anti-TH (AB152, Millipore), rabbit anti-pan-14-3-3 (sc-133233, Santa Cruz Biotech.), mouse anti-GAPDH (CSB-MA000195, Cusabio), rabbit anti-Vinculin (AB6039, Millipore), rabbit anti-SOD2 (HPA001814, Sigma-Aldrich), rabbit anti-Nrf2 (GTX103322, GeneTex), rat anti-HA (11867423001, Roche), rabbit anti-LC3B (NB100-2220, Novus Biologicals), rabbit anti-Cathepsin B (GTX101429, GeneTex), mouse anti-LAMP1 (sc-20011, Santa Cruz Biotech.), rabbit anti-αSyn MJFR1 (ab138501, Abcam), mouse anti-GFP (11814460001, Roche), mouse anti-β3-Tubulin (T8578, Sigma-Aldrich), mouse anti-GFAP-Alexa Fluor 488 (53-9892-82, Invitrogen), mouse anti-Syn-1 (610787, BD); goat anti-mouse-Alexa Fluor 488 (A11029, Invitrogen), goat anti-mouse-Alexa Fluor 568 (A11004, Invitrogen), goat anti-rabbit-Alexa Fluor 488 (A11034, Invitrogen), rabbit-Alexa Fluor 568 (A11036, Invitrogen).

### Mouse brain tissues

*Aldh1a1*^*−/−*^*/Aldh2*^−*/−*^ and wild-type mouse brain tissues were kindly provided by Prof. Randy Strong (UT Health San Antonio, Texas, USA). Mice with homozygous deletions of both *Aldh1a1* and *Aldh2* genes (double knock-out mice) on a C57BL/6 background were generated and genotyped as previously described [25]. Animals were maintained and experiments were conducted according to the National Institute of Health “Guide for the Care and Use of Laboratory Animals” and the approval by the Institutional Animal Care and Use Committee of the University of Texas Health Science Center at San Antonio. Five age-matched male *Aldh1a1*^*−/−*^*/Aldh2*^−*/−*^ and four wild-type mice were sacrificed between 11 and 12 months of age. No statistical methods were applied to choose the number of mice for each genotype; no randomization method was used to choose the animals for this analysis; no blind analysis was applied. Brains were rapidly collected following brief carbon dioxide anesthesia and decapitation. Midbrain and striatal tissues were dissected separately, then snap-frozen on dry ice and transferred to a –80 °C freezer for storage until assayed.

### Zebrafish

Wild-type zebrafish were staged and maintained according to standard procedures [26]. Embryos were obtained by natural mating and raised at 28.5 °C in Petri dishes containing fish water [50X: 25 g Instant Ocean (Aquarium systems, SS15-10), 39.25 g CaSO_4_, and 5 g NaHCO_3_ for 1 l] with a photoperiod of 13 h light/11 h dark. All zebrafish experiments were carried out at the Zebrafish Facility of the University of Padova (Italy). All experiments were performed on larvae before the free-feeding stage and did not fall under animal experimental laws according to EU Animal Protection Directive 2010/63/EU. Zebrafish larvae were randomly assigned to experimental groups and at least three independent experiments were carried out using about 25 individuals per group. No statistical methods were applied to choose the number of larvae for each condition; no blind analysis was applied. Larvae were treated with DMSO or 0.35 μM Disulfiram from 2 to 5 dpf, refreshing the treatment every 24 h in the morning, as previously described [14]. At 5 dpf, larvae were euthanized with 0.3 mg/ml tricaine added in fish water, then zebrafish heads were isolated by decapitation, frozen on liquid nitrogen and stored at –80 °C until assayed.

### Primary mouse neuron cultures

C57BL/6J wild-type mice were maintained and experiments were conducted according to the Italian Ministry of Health and the approval by the Ethical Committee of the University of Padova (Protocol Permit #690/2020-PR). Primary mouse cortical cell cultures were obtained from post-natal day 0 pups as previously described [14] and cultured in Neurobasal A medium (Life Technologies) supplemented with 2% v/v B27 Supplements (Life Technologies), 0.5 mM L-glutamine (Life Technologies), 100 U/mL penicillin, and 100 µg/mL streptomycin (Life Technologies). At 12 days in vitro (DIV12), cells were treated with 100 μM DOPAL for 24 h, then fixed in 4% paraformaldehyde (PFA) for immunofluorescence experiments.

### BE(2)-M17 cell lines maintenance, transfection, and treatments

Neuroblastoma-derived BE(2)-M17 cells (ATCC CRL-2267) were previously authenticated and characterized [27]. Cells were cultured in 50% of Dulbecco’s modified Eagle’s medium (DMEM, Voden) and 50% of F-12 Nutrient Mix (Voden), supplemented with 10% v/v fetal bovine serum (FBS, Corning) and 1% penicillin/streptomycin (Life technologies). When at 60% of confluency, cells were transiently transfected with 8xARE-GFP-SV40-BFP, HA-p62, HA-Ub or EGFP constructs using Lipofectamine 2000 (Invitrogen) with a DNA (µg): Lipofectamine (µl) ratio of 1:2. Cells were then treated and processed 24–48 h post transfection. Cellular treatments were performed in OptiMEM (Gibco) for the time and concentration indicated in each experiment. Serum starvation was induced by an overnight incubation with serum-free medium, followed by 2 h in Hank’s Balanced Salt Solution (HBSS, Life Technologies). The stable BE(2)-M17 cell lines with inducible dox-dependent αSyn overexpression was previously generated [14]. αSyn overexpression was induced by the addition of 100 ng/ml Doxycycline (dox) in the cell medium for 48 h before DOPAL treatment. Cell lines were tested for mycoplasma contamination by Hoechst staining and fluorescence imaging.

### Plasmids for mammalian cell expression

The plasmid encoding for HA-p62 for mammalian cell transient transfection was kindly gifted by Prof. Sandri (University of Padova). The pREP-8xARE-GFP-SV40-BFP (#134910) construct was purchased at Addgene. The pEGFP-N1 construct was purchased at Novagen.

### SDS-PAGE and western blot

BE(2)-M17 cells were harvested in RIPA Buffer (Cell Signaling Technology) supplemented with protease inhibitors cocktail (Roche) for 30 min in ice. Mouse brain tissues and zebrafish heads were homogenized in RIPA buffer as well. Lysates were clarified by centrifugation at 20,000× *g* at 4 °C for 30 min. Protein concentration in the cleared supernatant was determined using the Pierce® BCA Protein Assay Kit (Thermo Scientific) following the manufacturer’s instructions, and protein samples were loaded on gradient 4–20% Tris-MOPS-SDS gels (GenScript) or 8% poly-acrylamide Tris-Glycine-SDS gels. The resolved proteins were then transferred to PVDF membranes (BioRad), through a semi-dry Trans-Blot® Turbo™ Transfer System (BioRad). PVDF membranes were subsequently blocked in Tris-buffered saline plus 0.1% Tween (TBS-T) and 5% nonfat dry milk for 1 h at 4 °C and then incubated overnight at 4 °C with primary antibodies diluted in TBS-T plus 5% nonfat milk. After incubation with HRP-conjugated secondary antibodies (goat anti-rabbit-HRP, goat anti-mouse-HRP and rabbit anti-rat-HRP Sigma-Aldrich) at room temperature for 1 h, immunoreactive proteins were visualized using Immobilon® Classico Western HRP Substrate (Millipore) or Immobilon® Forte Western HRP Substrate (Millipore) by Imager CHEMI Premium detector (VWR). The densitometric analysis of the detected bands was performed by using the Fiji software.

### Immunofluorescence and confocal microscopy

BE(2)-M17 cells were fixed using 4% paraformaldehyde (PFA, Sigma-Aldrich) in PBS pH 7.4 for 20 min at room temperature. Cells were permeabilized in PBS-0.3% Triton-X for 5 min, followed by a 2-h saturation step in blocking buffer [1% Bovine serum Albumin (BSA) Fraction V, 2% FBS, 0.1% Triton-X and 50 mM Glycine in PBS]. Incubations with primary and secondary antibodies were performed in working solution (1:5 dilution of the blocking buffer) overnight at 4 °C or for 2 h at room temperature, following both incubations with three washing steps in working solution. For the nuclei staining, cells were incubated with Hoechst 33258 (Invitrogen, 1:2000 dilution in PBS) for 5 min. Confocal immunofluorescence z-stack images (1 µm step size) were acquired on the Zeiss LSM700 or Leica SP5 laser scanning confocal microscopes using a 63× oil immersion objective. Laser intensities were set right below the saturation threshold and kept unaltered for each group within the same experiment.

### Immunofluorescence image analysis

Confocal fluorescence images were processed and analyzed with the Fiji software (https://imagej.net/Fiji). Z-stacks images were converted to maximum intensity z-projections. The total p62, LAMP1, and αSyn fluorescence in each cell was measured as Mean Intensity in the region of interest (ROI). To analyze the p62 puncta count (normalized to cell area), fluorescence intensity, and size in each cell, a fluorescence threshold was set, and the Analyze Particle tool was used. For the quantification of p62- and LAMP1-positive puncta clustering, the distance among the *x, y* coordinates of each puncta centroid was measured by the Nearest Neighbor Distance (Nnd) plugin. For the activation of the Nrf2 reporter, the transfected cells were identified by the BFP. Once defined the ROI of the positive cells, the Mean Intensity of the GFP channel was normalized to the Mean Intensity of the BFP channel. The 8xARE-GFP-SV40-BFP positive cells were also analyzed for the Mean Intensity and number of puncta identified by the staining with the anti-p62 antibody. For the percentage of p62 and LAMP1 co-localizing puncta, the single channel images were converted to binary images (setting a threshold), and the ComDet v.0.4.2 plugin was used (4.00 pixels particle size, 20.00 intensity threshold, segmentation of larger particles).

### Real-time PCR

Untreated and 100 μM DOPAL-treated (for 18 h) cells from six-well plates at confluency were harvested and total RNA was extracted using the miRNeasy kit (217004, Qiagen) according to the manufacturer’s instructions. cDNA synthesis from 3.5 μg/sample of purified RNA were obtained using the SuperScript™ III Reverse Transcriptase kit (18080093, Thermo Fisher) following to the manufacturer’s instructions. *SQSTM1*, *NRF2*, and *MAP1LC3B* mRNA expression levels were measured by real-time PCR with SYBR Green technology in a CFX384 Touch-Real Time PCR Detection System (BioRad) and 10 ng of cDNA were amplified using the PowerUpTM SYBRTM Green Master Mix (#A25741, Thermo Fisher). The amplification protocol consisted of 50 °C for 2 min, then 95 °C for 2 min, followed by 40 cycles at 95 °C for 20 s and 60 °C for 1 min. *ACTB* gene was used as an internal standard in each sample. Threshold cycles (Ct) and melting curves were generated automatically by CFX384 Touch-Real Time PCR Detection System (BioRad). Relative gene expression levels were quantified using the comparative Ct method (2^–ΔΔCt^), analyzing each gene in triplicate from samples of three independent experiments. The following primer sequences were used for each gene:

*SQSTM1:*

Primer forward: 5’-AGACTACGACTTGTGTAGCGT-3’

Primer reverse: 5’-AAGGTGAAACACGGACACTTC-3’

*NRF2:*

Primer forward: 5’-GATGACTGCATGCAGCTTTTG-3’

Primer reverse: 5’-AGAGCCCAGTCTTCATTGCTA-3’

*MAP1LC3B:*

Primer forward: 5’-GAGAAGCAGCTTCCTGTTCTGG-3’

Primer reverse: 5’-GTGTCCGTTCACCAACAGGAAG-3’

*ACTB:*

Primer forward: 5’-GGCATCCTCACCCTGAAGTA-3’

Primer reverse: 5’-AGAGGCGTACAGGGATAGCA-3’.

### Protein pull-down assay with APBA resin

The isolation of catechol-modified proteins was achieved by protein pull-down assay with m-Aminophenylboronic acid (APBA)—agarose resin (A8312, Sigma-Aldrich) as previously described [13]. Briefly, 25 µl of APBA resin were washed in distilled water and equilibrated in PBS. For each washing step, supernatants were discarded after centrifugation at 10,000× *g* for 2 min. The resin was then incubated with 100 µg of proteins from BE(2)-M17 cell lysates in a total volume of 600 µl of RIPA buffer supplemented with protease inhibitor cocktail and 1 mg/ml NaCNBH_3_, overnight at 4 °C on rocker. The following day, the resin was washed twice in PBS/acetonitrile (50:50) and finally in distilled water. Proteins were then collected from the resin by incubating 20 μl of Laemmli buffer 4×, loaded into 4–20% gradient SDS-PAGE and compared with the total lysate by western blot.

### Immunoprecipitation of HA-tagged proteins

HA-tagged proteins were immunoprecipitated using Pierce anti-HA magnetic beads (88836, Thermo Scientific) according to the manufacturer’s instructions. Briefly, 500 µg of proteins from BE(2)-M17 cell lysate were diluted to a final volume of 600 µl of RIPA buffer supplemented with protease inhibitors cocktails and 1 mg/ml NaCNBH_3,_ and incubated with 50 µl of anti-HA magnetic beads for 2 h at room temperature. The unbounded proteins were then removed by the isolated HA-tagged proteins by a magnetic rack and the flow-through was collected separately. After washing with TBS-T and distilled water, HA-tagged proteins were eluted by incubation with Laemmli buffer 4× for 10 min at 95 °C.

### Electron microscopy of p62 oligomers

After immunoprecipitation from untreated and DOPAL-treated BE(2)-M17 cells expressing HA-p62 proteins isolated as described above, samples were eluted by incubation with 200 µl of 2 mg/ml HA peptide (Peptide Synthesis Facility, UNIPD) for 20 min at 37 °C on the rocker. Based on the initial volume of anti-HA magnetic beads used, their concentration and binding capacity, elution was diluted to the final concentration of 10 μg/μl for further analysis by transmission electron microscopy (TEM) and immunogold staining. Briefly, 30 µl of proteins were let absorbing on grids for 5 min at room temperature, following a blocking step in 0.5% BSA in PBS. Anti-p62 primary antibody (ab109012, Abcam) was then incubated for 30 min to a 1:50 dilution in PBS, following three washes in PBS and incubation with goat anti-rabbit IgG coupled to gold particles of 5-nm diameter (Sigma-Aldrich) diluted 1:100 in blocking solution for 30 min at room temperature. After extensive washes in PBS and water, grids were negatively stained with 1% Uranyl Acetate for 2 min at room temperature. Immunolabeled grids were observed with a Tecnai G2 (FEI) transmission electron microscope operating at 100 kV. Images were captured with a Veleta (Olympus Soft Imaging System) digital camera and analyzed with Fiji software. Distances (nm) among gold particles were analyzed with the nearest neighbor distance (Nnd) plugin and expressed as frequency distribution.

### Mass spectrometry analysis of DOPAL-modified p62

HEK293T cell were cultured in DMEM (Voden) supplemented with 10% FBS (Corning) and 1% Penicillin/Streptomycin (Life Technologies) in a 15 cm-diameter dish. Cells were transfected with 40 μg of HA-p62 cDNA using Polyethylenimine (23966-1, Polysciences Inc) a DNA(µg):Lipofectamine(µl) ratio of 1:3. Seventy-two hours post transfection, total cell lysates was incubated with 150 μl of anti-HA magnetic beads for immunoprecipitation as described above. After extensive washes in TBS-0.1% Tween, samples were resuspended in 150 μl of H_2_O. 10% of the immunoprecipitation was eluted and analyzed in SDS-PAGE with a standard curve of BSA for quantification. Once the concentration of HA-p62 molecules on beads was determined (2 μM), 200 μM DOPAL was incubated overnight at 25 °C under constant shaking (350 rpm). After the addition of 1 mg/ml NaCNBH_3_ for 30 min, the supernatant was removed and replaced with distilled water. 10% of the reaction was collected and eluted for further analysis by near-infrared fluorescence (nIRF) detection by scanning the membrane at the LiCor Odissey using the 800 nm filter as previously described [14], SDS-PAGE and staining with colloidal Coomassie or immunoblot with the anti-p62 antibody. The remaining reaction was then analyzed by mass spectrometry. In details, the anti-HA magnetic beads with the immunoprecipitated proteins, untreated or DOPAL-treated, were resuspended in 130 µl of 50 mM ammonium bicarbonate, reduced with 10 mM dithioerithreitol (DTE) at 56 °C for 30 min, alkylated with 55 mM iodoacetamide at room temperature for 20 min in the dark and finally divided into two aliquots. The first one was digested with chymotrypsin, the second one with trypsin, both in a ratio of 1:50 (w/w) at 37 °C overnight, with shaking (600 rpm). The day after, the peptides were desalted using stage tip C18 columns, resuspended in 10 µl of 10% formic acid and injected in nUPLC-MS/MS, using the Q-Exactive mass spectrometer (Thermo Scientific, Brema, Germany) equipped with nano-elettrospray source (Proxeon Biosystems) and nUPLC Easy-nLC 1000 (Proxeon Biosystems). Peptide separations occurred on a homemade (75 µm i.d., 15 cm long) reverse phase silica capillary column, packed with 1.9 µm ReproSil-Pur 120 C18-AQ (Dr. Maisch GmbH, Germany). A gradient of eluents A (distilled water with 0.1% v/v formic acid) and B (acetonitrile with 0.1% v/v formic acid) was used to achieve separation (300 nL/min flow rate), from 2% B to 40% B in 88 min. Full scan spectra were acquired with the lock-mass option, resolution set to 70,000 and mass range from *m/z* 300 to 2000. The ten most intense doubly and triply charged ions were selected and fragmented. All MS/MS samples were analyzed using Mascot (version 2.6, Matrix Science) search engine to search the human proteome 20220223 (101,038 sequences; 40,994,391 residues), in which the sequence of HA-p62 was added. Searches were performed with the following settings: trypsin or chymotrypsin as proteolytic enzyme; 2 missed cleavages allowed; carbamidomethylation on cysteine as variable modification; protein N-terminus-acetylation, methionine oxidation, DOPAL modification as Schiff base/Schiff base reduced by NaBH_3_CN in their catechol and quinone forms on lysine residues and protein N-terminus, DOPAL modification as catechol/catechol-hydrated/quinone/quinone-hydrated on lysine, protein N-terminus and cysteine residues as variable modifications; mass tolerance was set to 5 ppm and to 0.02 Da for precursor and fragment ions, respectively. Raw data were also analyzed using the MaxLynx workflow within the software MaxQuant v 2.1.4.0, with the following parameters: 3 missed cleavages allowed; carbamidomethylation on cysteine as variable modification; protein N-terminus-acetylation, methionine oxidation, DOPAL modification as Schiff base/Schiff base reduced by NaBH3CN in their catechol and quinone forms on lysine residues and protein N-terminus, DOPAL modification as catechol/catechol-hydrated/quinone/quinone-hydrated on lysine, protein N-terminus and cysteine residues as variable modifications within the non-cleavable cross-linked peptide search; mass tolerance was set to 5 ppm and peak refinement option was selected. In order to reduce the computational time, a FASTA file containing the only HA-p62 sequence was used as protein database.

### Measurement of lysosomal pH

Lysosomal pH was measured as previously described [28]. Briefly, BE(2)-M17 cells were plated in Lumox 96 multiwell (Sarstedt) and treated with 100 µM DOPAL or 50 µM Chloroquine for 18 h once reached 80% of confluency. Cells were then rinsed once in PBS and treated with the ratiometric dye, 2-(4-pyridyl)-5-((4-(2-dimethylaminoethylaminocarbamoyl) methoxy) phenyl) oxazole (RatioWorks PDMPO, AAT Bioquest) at a final concentration of 2 μM in OPTIMEM for 5 min in the dark. After the incubation, cells were rinsed three times in OPTIMEM and fluorescence was measured at 37 °C using a multi-plate reader (EnVision, Perkin Helmer). Fluorescence emission at 535 nm was acquired upon excitation at 340 nm and 380 nm and the ratio was calculated.

### Statistical analysis

The statistical analysis of the collected data, as well as the proper graphical visualization, were performed by GraphPad Prism Software Inc. (version 7). In general, data were collected from at least *n* = 3 independent experiments, with multiple technical replicates for each group. No statistical methods were applied to choose sample size as it was not possible to determine the expected effect size a priori. Randomization was used when suitable with the experimental setup, that is treatments were randomly assigned to cellular cultures and zebrafish individuals. Blinding was not used for data collection. Data analysis was conducted by algorithms and automated softwares to be as unbiased as possible. Within each biological replicate, data were normalized to the mean value of the untreated condition and plotted as fold-change variations of the treated samples compared to controls. Data from independent experiments were then pooled together and represented as mean ± SEM displaying each individual point. Outliers were only excluded when data differed from the mean value as twice the standard deviation using the Graphpad statistical analysis tool. The datasets were analyzed without normality assumption, hence nonparametric tests were applied. The statistical analysis among mean values was performed by unpaired *t* test with Welch’s correction (datasets from in vivo models), by two-tailed nonparametric Mann–Whitney test (two datasets), by the two-tailed nonparametric Kruskall–Wallis test with the Dunn’s multiple comparisons test (more than two datasets) or by Two-way ANOVA with Sidak’s multiple comparison test. For datasets with no variability in the untreated group, the one-sample *t* test was performed against the theoretical mean =1. Statistical significance was defined for *P* value < 0.05 (*P < 0.05, **P < 0.01, ***P < 0.001, ****P < 0.0001).

## Results

### DOPAL buildup leads to p62 accumulation and oligomerization

**Fig. 1:**
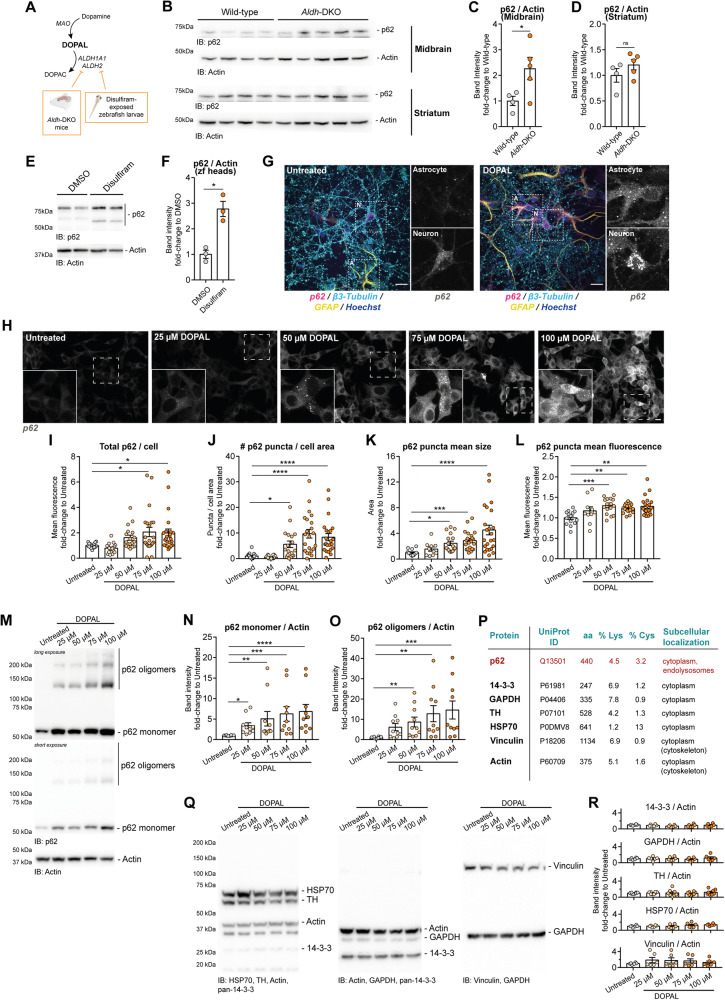
DOPAL buildup leads to p62 accumulation and oligomerization in vivo and cellular models. **A** Schematic representation of dopamine catabolic pathway and strategies to induce DOPAL buildup in mouse and zebrafish in vivo models. **B** Western blot analysis of p62 in midbrain and striatal brain tissues from wild-type (*n* = 4) and *Aldh*-DKO (*n* = 5) 12-month-old mice. Relative quantification of p62 levels normalized to Actin **C** in the midbrain and **D** in the striatum. Data are normalized to the wild-type and analyzed by unpaired t test with Welch’s correction (*P < 0.05). **E** Western blot analysis of p62 in the protein lysates extracted from the heads of zebrafish larvae exposed to DMSO or 0.35 μM Disulfiram from 2 to 5 dpf. **F** Relative quantification of p62 levels normalized to Actin and to DMSO samples, and analyzed by ratio paired *t* test (*P < 0.05). **G** Immunofluorescence of p62 (magenta) in primary mouse cortical cell cultures in untreated and 100 μM DOPAL-treated cells for 24 h. Neurons and astrocytes are identified by β3-Tubulin (cyan) and GFAP (yellow) markers, respectively. Nuclei are stained by Hoechst (blue). Scale bar: 10 μm. In the inset, higher magnifications of the p62 staining in the two cell types. **H** Immunofluorescence of p62 cellular distribution in BE(2)-M17 cells treated with increasing DOPAL concentrations (0-25-50-75-100 µM) for 18 h. Scale bar: 10 µm. Relative quantification of **I** total p62/cell (expressed as mean fluorescence intensity), **J** number of puncta/cell area (μm^2^), **K** mean puncta size (μm^2^), and **L** mean puncta fluorescence. **M** In the same experimental conditions, the immunoblot with the anti-p62 antibody reveals a DOPAL dose-dependent accumulation of p62 **N** monomer and **O** oligomeric forms, after normalization to Actin used as loading control. **P**, **Q** In the table, the UniProt ID, the number of total amino acids, the percentage of lysine and cysteine residues in the sequence and the subcellular localization of proteins analyzed by western blot in the same experimental conditions, namely 14-3-3 proteins, GAPDH, TH, HSP70, Vinculin, and Actin. **R** The quantification, after normalization to Actin used as a loading control, does not reveal any significant DOPAL-dependent variation in the diverse cellular proteins. **I**–**L**, **N**, **O**, **R** Data are pooled together from three to five independent experiments normalized to each untreated sample, and analyzed by Kruskall–Wallis test with Dunn’s multiple comparison test (*P < 0.05, **P < 0.01, ***P < 0.001, ****P < 0.0001).

We previously demonstrated that in vivo models of impaired DOPAL detoxification display αSyn accumulation in the brain, dopaminergic neuron loss and a parkinsonian motor phenotype (Fig. 1A) [14, 25]. Here, we observed that in the midbrain of 12-month-aged double knock-out mice lacking the cytosolic ALDH1A1 and mitochondrial ALDH2 (*Aldh*-DKO mice), p62 was significantly accumulated as compared to littermate wild-type mice, with a similar trend, although non-significant, in the striatum (Fig. 1B–D). Similarly, zebrafish larvae exposed to the ALDH inhibitor disulfiram (0.35 μM disulfiram from 2 to 5 dpf), presented a significant increase of p62 in their brain (Fig. 1E, F). At the cellular level, the accumulation of p62 cytosolic puncta appeared in the soma of both neuronal cells and astrocytes when 100 µM DOPAL was exogenously administered to primary mouse cultures for 24 h (Fig. 1G).

To gain insights into the molecular mechanisms of DOPAL-induced p62 cellular buildup, we moved to neuroblastoma-derived BE(2)-M17 cells, a catecholaminergic in vitro model easier to manipulate experimentally [27]. We initially studied by immunofluorescence the effect of increasing doses of DOPAL (0, 25, 50, 75, and 100 µM for 18 h) on p62. As shown in Fig. 1H, we detected a dose-dependent p62 accumulation and clustering upon DOPAL treatment, with a significant increase in the number of cytosolic puncta, as well as puncta size and puncta fluorescence, with 50 µM DOPAL treatment as minimum threshold for statistical significance (Fig. 1I–L). Then, we analyzed p62 levels in protein lysates extracted from DOPAL-treated cells by western blot, revealing a dose-dependent buildup of both p62 monomer and SDS-resistant multimeric species (Fig. 1M–O). The comparison of the DOPAL effect on the two species highlighted a more distinct impact on p62 oligomers (up to 15-fold increase on average), which is coherent with the accumulation of p62-positive puncta observed by immunofluorescence. We then sought to determine whether DOPAL-induced accumulation and cross-linking affected other highly expressed and ubiquitous cellular proteins or was specific to p62. Under the same experimental conditions, cytosolic proteins like 14-3-3s, glyceraldehyde-3-phosphate dehydrogenase (GAPDH), tyrosine hydroxylase (TH), heat-shock protein-70 (HSP70) or vinculin did not significantly accumulate nor oligomerize upon DOPAL treatment (Fig. 1P–R), despite presenting a cytosolic localization, a comparable fraction of cysteine and lysine residues in their sequence or having been reported to be targets of DOPAL modification in previous in vitro studies (i.e., GAPDH, TH) [29, 30].

### DOPAL-induced Nrf2 activation and oxidative stress contribute to p62 buildup

**Fig. 2:**
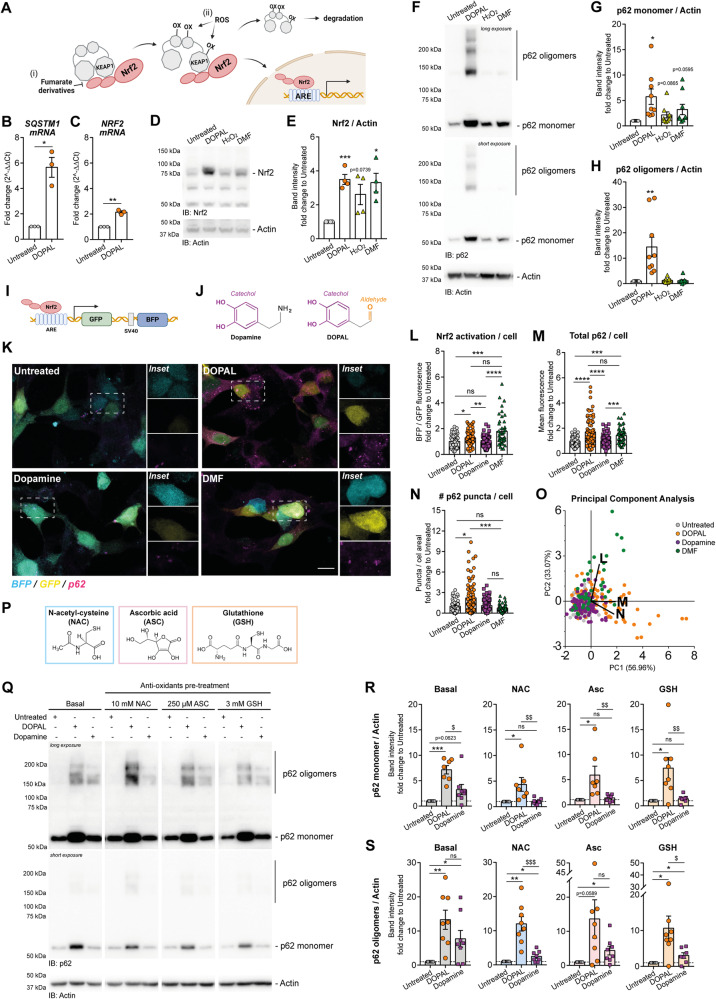
DOPAL-induced Nrf2 pathway activation and oxidative stress may contribute to p62 buildup. **A** Schematic representation of Nrf2 activation by oxidative stress and DMF. The (i) direct binding of fumarate derivatives as well as (ii) cysteines oxidation following oxidative stress promote Keap1 detachment from Nrf2, which then translocates in the nucleus and binds to ARE. Real-time PCR of **B** *SQSTM1* and **C** *NRF2* mRNA levels in untreated and 100 µM DOPAL for 18 h. Data are pooled together from three independent experiments normalized to each untreated sample, and analyzed by one-sample *t* test (*P < 0.05, **P < 0.01). **D** Immunoblot of Nrf2 levels in BE(2)-M17 cells after treatments with 100 µM DOPAL, 10 µM H_2_O_2_ and 10 µM DMF for 18 h, and the untreated control. **E** Relative quantification after normalization to Actin used as loading control. Data are pooled together from three independent experiments normalized to each untreated sample, and analyzed by one-sample *t* test (*P < 0.05, ***P < 0.001). **F** Immunoblot of p62 in the same conditions and relative quantification of **G** p62 monomer and **H** p62 oligomers after normalization to Actin used as loading control. Data are pooled together from six independent experiments normalized to each untreated sample, and analyzed by one-sample *t* test (*P < 0.05, **P < 0.01). **I** Schematic representation of the Nrf2-ARE reporter. In transfected cells, the BFP is constitutively expressed while GFP expression depends on Nrf2 activation and binding to the 8xARE sequence. **J** Comparison of the molecular structures of dopamine and DOPAL. Both molecules have the catechol moiety (in purple) but only DOPAL has the aldehyde (in orange). **K** Representative confocal images of BE(2)-M17 cells transfected with Nrf2-ARE reporter and treated with 100 µM DOPAL, 100 µM Dopamine and 10 µM DMF for 18 h, as well as untreated cells. The constitutively expressed BFP (cyan) and the GFP (yellow) expressed under the activation of Nrf2 in the transfected cells are displayed as an overlay with the immunostaining for the endogenous p62 (magenta). Relative quantification of **L** Nrf2 pathway activation expressed as BFP/GFP fluorescence/cell, **M** total p62/cell measured as mean fluorescence intensity, **N** number of p62 puncta/cell. Data are collected form three independent experiments, normalized to the untreated samples, and analyzed by Kruskall–Wallis test with Dunn’s multiple comparison test (*P < 0.05, **P < 0.01, ***P < 0.001, ****P < 0.0001). **O** In the same experiment, principal component analysis of the three variables quantifies for each cell, where vectors (**L**, **M**, **N**) correspond to each quantification. **P** Molecular structures of antioxidants molecules N-Acetyl-cysteine (NAC), Ascorbic acid (ASC) and glutathione (GSH) used to prevent oxidative stress. **Q** Immunoblot with the anti-p62 antibody in the lysates of BE(2)-M17 treated for 18 h with 100 µM DOPAL, 100 µM Dopamine and the untreated cells. Prior to catechols administration, cells were pre-treated with 10 mM NAC, 250 µM ASC or 3 mM GSH for 1 h and then washed out. Quantification of **R** p62 monomer and **S** p62 oligomers in the lysates (after normalization to Actin used as loading control) of BE(2)-M17 treated with DOPAL or dopamine after the exposure to each antioxidant molecules. Data are pooled together from four independent experiments normalized to each untreated sample, and analyzed by one-sample *t* test (*P < 0.05, ***P < 0.001) and Mann–Whitney test (^$^*P* < 0.05, ^$$^*P* < 0.001, ^$$$^*P* < 0.0001). **A**, **I** Artwork created with BioRender.com.

Based on these observations, we then asked whether p62 protein buildup derived from an increase in expression of its gene *SQSTM1*, which is known to be regulated by the nuclear factor erythroid 2-related factor (Nrf2) [31]. Both oxidative stress and cell metabolism can activate the Nrf2-Keap1 pathway [32], in which the direct binding of fumarate derivatives as well as cysteines oxidation promote Keap1 dissociation from Nrf2. The latter then translocates into the nucleus and binds to Antioxidant-Response Elements (AREs), thus promoting the transcription of several genes, including *SQSTM1* (Fig. 2A). As the catechol moiety of DOPAL is prone to autoxidation generating reactive oxygen species (ROS) [33], we evaluated whether DOPAL-induced redox signaling could be responsible for *SQSTM1* mRNA and protein accumulation. qPCR analysis of DOPAL-treated BE(2)-M17 cells confirmed a significant increase in both *SQSTM1* and *NRF2* mRNA (Fig. 2B, C). Coherently, Nrf2 protein levels increased in 100 µM DOPAL-treated cells, as observed for 10 µM dimethyl fumarate (DMF) and 10 µM H_2_O_2_, used as positive controls of Nrf2-Keap1 pathway activation (Fig. 2D, E). Under the same conditions of DOPAL, DMF, and H_2_O_2_ treatment, p62 monomer displayed a similar trend toward increasing amounts, suggesting that DOPAL enhances Nrf2 and p62 protein levels through a mechanism that might be, at least in part, dependent on ROS-mediated increased Nrf2 transcriptional activity (Fig. 2F, G). However, a significant p62 oligomerization was only observed for DOPAL-treated cells and devoid in either DMF- or H_2_O_2_-stimulated cells (Fig. 2H).

To gain further mechanistic insights, we used the pREP-8xARE-GFP-SV40-BFP construct that acts as reporter for Nrf2-ARE axis activation [34]. Briefly, in transfected cells, the BFP is constitutively expressed while GFP expression depends on Nrf2 dissociation from Keap1 and binding to the 8xARE sequence (Fig. 2I). Hence, we compared the degree of Nrf2-induced GFP transcription (normalized by BFP fluorescence in each transfected cell) and the p62 levels and puncta formation in untreated, 100 µM DOPAL- and 10 µM DMF-treated cells (Fig. 2K). We also treated the cells with 100 µM dopamine, which shares the same catechol group of DOPAL but lacks the aldehyde moiety that is present only on DOPAL structure (Fig. 2J). This experiment is conceived to dissect whether the Nrf2 pathway upregulation depends on the catechol oxidation to quinone and is associated with ROS generation. As shown in Fig. 2L–N, both DMF and, at lesser extent, DOPAL induced a significant activation of the Nrf2 reporter, accompanied by increased total p62 as compared to untreated and dopamine-treated cells, supposedly due to increase p62 transcription (Fig. 2L–M). However, only DOPAL-treated cells presented consistent p62 cytosolic puncta formation (Fig. 2N). To analyze the correlation between the three read-outs (Nrf2 activation, total p62, and p62 puncta) for each cell in the four conditions, we applied a principal component analysis (PCA). As shown in Fig. 2O, the principal component 1 (PC1) corresponding to total p62 (vector M) accounted for almost 57% of the variability, with a positive correlation in DOPAL- and DMF-treated cells. While only DMF-treated cells positively correlated with Nrf2 activation along vector L, (with PC2 accounting for 33% of the variability), DOPAL-treated cells rather clustered for the accumulation of p62 cytosolic puncta (along vector N, with a positive correlation for PC1 and a negative correlation for PC2). Of note, dopamine-treated cells did not contribute to the variability in the PCA analysis, as they essentially overlapped with the untreated cells.

To further understand the potential contribution of the oxidation of DOPAL catechol moiety and Nrf2 activation on p62 proteostasis, we tested whether an antioxidant cellular environment could prevent p62 accumulation and oligomerization. To this aim, we performed a pre-treatment with high concentrations of antioxidants molecules, namely 10 mM N-acetylcysteine (NAC), 250 µM ascorbic acid (Asc) and 3 mM glutathione (GSH), which were previously shown to modulate DOPAL reactivity by reducing catechol oxidation [35, 36] (Fig. 2P, Q). Following a 1-h pre-treatment to allow the three antioxidant molecules to diffuse in the cells, cell medium was washed out before the addition of DOPAL or dopamine, to avoid any reaction between the catechols and residual antioxidant compound in the extracellular space. Although an antioxidant cellular environment displayed a tendency to reduced accumulation of monomeric p62 caused by both DOPAL and dopamine treatment, DOPAL effect on p62 oligomerization was not affected by these compounds, while dopamine-induced p62 oligomerization was evidently reduced (Fig. 2R, S).

Overall, these data suggested that, while DOPAL-derived oxidative stress contributes to Nrf2 pathway activation with consequent increased expression of p62, the dramatic effect observed on p62 oligomerization mostly depends on an additional mechanism related to the specific reactivity of DOPAL on p62.

### DOPAL covalently modifies p62 generating protein cross-linking

**Fig. 3:**
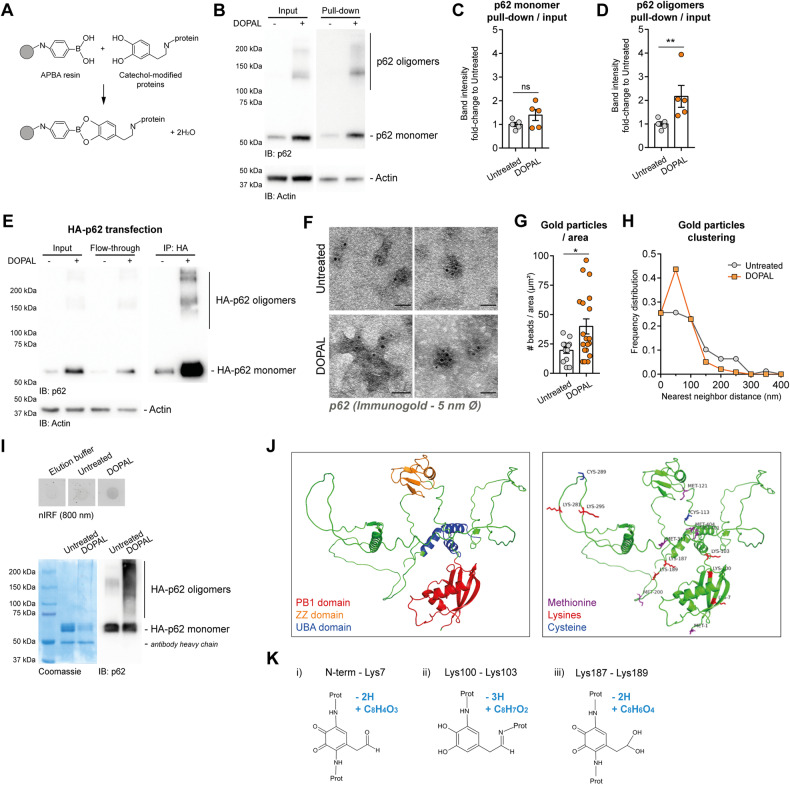
DOPAL covalently modifies p62-generating protein cross-linking. **A** Schematic representation of chemical isolation of catechol-modified proteins by the boronic group of APBA resin. **B** Protein pull-down assay with APBA resin in BE(2)-M17 untreated and 100 µM DOPAL-treated cells for 18 h. The immunoblot with the anti-p62 antibody shows the DOPAL-induced p62 accumulation and oligomerization in the input, and p62 enrichment in the DOPAL-modified protein fraction. Relative quantification of **C** p62 monomer and **D** p62 oligomers expressed as ratio between the pull-down fraction and the input. Data are pooled together from three independent experiments, normalized to each untreated sample and analyzed by Mann–Whitney test (**P < 0.01). **E** Immunoprecipitation with anti-HA magnetic beads of lysates of BE(2)-M17 transfected with HA-p62, untreated and 100 µM DOPAL-treated for 18 h. The immunoblot with the anti-p62 antibody shows the DOPAL-induced p62 accumulation and oligomerization in the input and in the IP. **F** Relative EM micrographs of the IP elution with 2 mg/ml HA peptide, after immunogold staining with the anti-p62 antibody and anti-rabbit secondary antibody conjugated with 5 nm-diameter gold nanoparticles. Scale bar: 100 nm. **G** In the electron micrographs, number of gold particles per area unit (μm^2^) analyzed by Mann–Whitney test (*P < 0.05). **H** Quantification of the Nearest Neighbor Distance (Nnd) among gold particles in the EM micrographs. The Nnd (nm) is expressed as frequency distribution in the both untreated and DOPAL-treated conditions. **I** Immunoprecipitation of HA-p62 from HEK293T transfected cells and on-beads DOPAL modification upon incubation with 200 µM DOPAL for overnight. The nIRF detection at 800 nm in the elution highlights the DOPAL modification on the treated protein, while the Coomassie stained SDS-PAGE, as well as the immunoblot, display the generation of DOPAL-induced cross-linking among p62 molecules in high-molecular-weight species. **J** On the left, secondary structure of p62 domains obtained by AlphaFold (AF-Q13501-F1) with the PB1 domain, the ZZ domain and the UBA domain highlighted in red, orange and blue, respectively. Upon HA-p62 on-beads incubation with DOPAL, MS/MS analysis identified four residues (Cys113, Lys281, Cys289, Lys295,) modified by the catechol moiety of DOPAL, Lys281, and Lys295 modified through Schiff base and few residues modified to give rise loop-linked peptides (Protein N-terminus–Lys7; Lys100–Lys103; Lys187–Lys189). The amino acidic numbering refers to the p62 sequence without the HA tag. On the right, in the same p62 secondary structure, the above relevant residues are highlighted: lysines in red, cysteines in blue, six DOPAL-induced oxidized methionines in purple. **K** Schematic representation of the DOPAL reactions occurring in the formation of loop-linked peptides: (i) Lys–Lys (or N-term) via Michael addition on the quinone; (ii) Lys–Lys (or N-term) via Michael addition on the catechol and Schiff base on the aldehyde; (iii) Lys–Lys (or N-term) via Michael addition on the hydrated quinone. In light blue, the chemical composition of Δ mass introduced on the molecular mass of peptides upon each reaction.

We hypothesized that DOPAL-induced p62 oligomerization may be the consequence of a covalent modification of p62 cysteines and lysines by DOPAL, thus generating protein cross-linking and aggregation. To test this hypothesis, we first performed a protein pull-down assay with the aminophenylboronic acid (APBA) resin, which has been used to isolate catechol-modified proteins [8, 13] (Fig. 3A). While p62 monomer showed only a tendency toward accumulation in the pull-down of DOPAL-treated cells, p62 oligomers were significantly enriched in the isolated fraction (Fig. 3B–D). We then setup an immunoprecipitation assay of p62 in BE(2)-M17 cells transiently transfected with HA-tagged p62, using anti-HA magnetic beads (Fig. 3E). After elution with an excess of HA peptide (2 mg/ml), we analyzed the isolated proteins by transmission electron microscopy (TEM) after immunogold labeling using the anti-p62 primary antibody and anti-rabbit secondary antibody conjugated with 5 nm-diameter gold nanoparticles (Fig. 3F). Despite no defined recursive structures were evident in the electron micrographs, there was a substantial increase in p62-associated gold particles per unit of area in the DOPAL-derived sample (Fig. 3G), along with reduced nearest neighbor distances (Nnd) among beads analyzed by frequency distribution, indicating a DOPAL-dependent higher clustering of p62 molecules in multimeric aggregates (Fig. 3H).

To map the DOPAL-modification site(s) on p62 sequence, we isolated HA-p62 by immunoprecipitation (IP) from transfected HEK293T cells, which display a higher transfection efficiency than BE(2)-M17 cells, thus providing sufficient protein for mass spectrometry (MS). Once the flow-through was removed and washed extensively, HA-p62 was incubated with 200 μM DOPAL while still bound to the magnetic beads to perform a direct modification assay (see Methods for further details). After an overnight incubation following reduction by 1 mg/ml NaCNBH_3_, 10% of the reaction was eluted and analyzed by biochemical approaches (Fig. 3I). Of note, the detection of the near-infrared fluorescence (nIRF) derived from oxidized catechols bound to proteins [37] revealed an increased signal in the DOPAL-incubated sample. To further support a direct modification, both Coomassie staining and immunoblot showed a consistent enrichment in high-molecular-weight p62 species. The remaining reactions (+/− DOPAL) bound to magnetic beads were then incubated with trypsin and chymotrypsin, and liquid chromatography coupled to mass spectrometry (LC-MS/MS) was performed on the digested HA-p62 products. The use of the two enzymes with different proteolytic specificity allowed to obtain an overall sequence coverage of 73% for the untreated sample and 95% for the DOPAL-treated reaction. While in the untreated sample, only one methionine (Met200) was found partially oxidated, the MS/MS analysis of the DOPAL reaction revealed several partially oxidized methionine residues (Met1, Met121, Met200, Met311, Met401, Met404, highlighted in purple in the modeling of p62 structure predicted by AlphaFold (AF-Q13501-F1, Fig. 3J). More interestingly, two cysteines and two lysines (out of 14 and 20 in p62 sequence, respectively) were modified by DOPAL in Michael addiction via the catechol moiety. These residues were identified by the MS/MS analysis as Cys113 and Cys289 shown in blue, Lys281 and Lys295 shown in red in Fig. 3J. From a structural point of view, the four residues are accessible to DOPAL modification as they map within an unstructured loop between the PB1 and the ZZ domains (Cys113) or within the intrinsically unfolded domain in the central region of the protein (Lys281, Cys289, Lys295). The two lysine residues (Lys281 and Lys295) were also modified in the Schiff base via the aldehyde moiety of DOPAL. In order to define the amino acidic residues involved in the formation of p62 oligomers, the mass spectrometry data were further analyzed using the MaxLynx workflow, integrated into the MaxQuant environment [38]. In particular, the presence of peptides, which were cross-linked through cysteine and/or lysine residues, could be then assessed by matching experimental MS and MS/MS data of tryptic digests. Using this approach, the protein N-terminus and five extra lysines were identified as modification sites by DOPAL. In particular, a loop-linked peptide was identified between the protein N-terminus and Lys7, through two Michael additions on the quinone form of DOPAL. Similarly, a loop-linked peptide was identified between Lys100 and Lys103, through a Michael addition on the catechol moiety and a Schiff base formation on the aldehydic moiety of DOPAL. Finally, Lys187 and Lys189 were loop-linked through two Michael additions on the hydrated quinone form of DOPAL (Fig. 3K).

Taken together, these data support the reactivity of DOPAL towards p62, resulting in covalent modification of exposed cysteines and lysines and protein cross-linking.

### DOPAL affects the role of p62 in the autophagic pathway

**Fig. 4:**
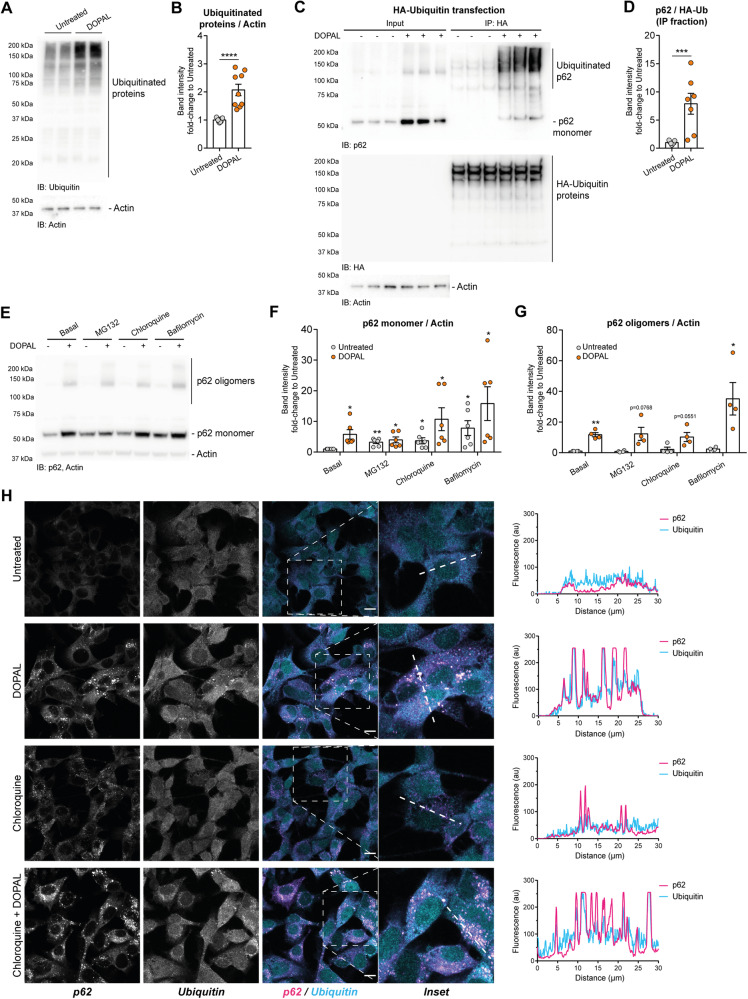
DOPAL promotes p62 ubiquitination. **A** Immunoblot with the anti-ubiquitin antibody in BE(2)-M17 cells treated with 100 µM DOPAL for 18 h and the untreated control. **B** Relative quantification of total ubiquitinated proteins after normalization to Actin used as a loading control. Data are pooled together from four independent experiments normalized to each untreated sample and analyzed by Mann–Whitney test (****P < 0.0001). **C** Immunoprecipitation of ubiquitinated proteins in BE(2)-M17 expressing HA-Ubiquitin and treated with 100 µM DOPAL for 18 h. The image displays the immunoblot with the anti-p62, anti-HA, and anti-Actin antibodies in the input and the immunoprecipitation. **D** Quantification of the fraction of p62 in the immunoprecipitation, normalized to the ubiquitinated proteins in this fraction. Data are pooled together from three independent experiments normalized to each untreated sample and analyzed by Mann–Whitney test (***P < 0.001). **E** Western blot of p62 in BE(2)-M17 in untreated and 100 µM DOPAL-treated cells, both in basal conditions and after 20 µM MG132, 50 µM chloroquine and 20 nM Bafilomycin treatments for 18 h. Relative quantification of **F** p62 monomer and **G** p62 oligomers after normalization to Actin used as loading control. Data are pooled together from three independent experiments normalized to each untreated sample, and analyzed by one-sample *t* test (*P < 0.05, **P < 0.01) relative to basal-untreated condition. Statistical analysis by two-way ANOVA also reveals a significant effect of DOPAL treatment on both p62 monomer (**P < 0.01) and p62 oligomers (***P < 0.001). **H** Immunofluorescence of p62 cytosolic distribution (magenta in the overlay) and ubiquitin (cyan in the overlay) in untreated and 100 µM DOPAL-treated cells, both in basal conditions and after 50 µM chloroquine treatment for 18 h. Scale bar: 10 µm. On the right side, the graphs display the co-localization between p62 and ubiquitin fluorescence signals along the dotted lines marked in the insets.

Having demonstrated that DOPAL buildup increases p62 oligomerization by a direct reaction, we then investigated the functional consequences on cellular proteostasis. We previously showed that DOPAL buildup results in a significant alteration of cellular proteostasis in primary neurons [14]. Consistently, 100 μM DOPAL treatment for 18 h in BE(2)-M17 cells induced a significant accumulation of ubiquitinated proteins as detected by western blot, suggesting dysfunctional protein quality control (Fig. 4A, B). Based on the key role of p62 at the crossroads of the proteasome and the autophagy-lysosomal pathways [39], we sought to investigate the consequences of DOPAL-induced oligomerization in p62 function and clearance.

We first immunoprecipitated ubiquitinated proteins from HA-Ubiquitin expressing BE(2)-M17 cells and detected a consistently higher p62 signal in the IP fraction of DOPAL-treated cells, suggesting either increased p62 poly-ubiquitination and/or increased interaction with ubiquitinated proteins (Fig. 4C, D). We then measured the accumulation of p62 upon blocking either the proteasome by 20 μM MG132, or the autophagic pathway by 50 μM chloroquine or 20 nM bafilomycin, with or without 100 μM DOPAL (Fig. 4E). All tested conditions resulted in a significant buildup of p62 monomer and DOPAL-induced oligomers, with more pronounced effects when autophagy was inhibited (Fig. 4F, G). Hence, we focused our attention on the trafficking of p62 along the autophagic components. From the immunofluorescence study of p62 and ubiquitin in untreated and DOPAL-treated cells, and in combination with autophagy blockage by chloroquine, it emerged that the DOPAL-induced p62 cytosolic puncta strongly co-localized with ubiquitin-positive puncta (Fig. 4H). In the first steps of autophagic degradation, p62 recognizes and interacts with ubiquitinated proteins via the C-terminal UBA domain, thus we hypothesized an upregulation of this degradation pathway as an attempt to promote the clearance of aberrant proteins accumulated by DOPAL. The treatment with chloroquine alone did not result in high immunofluorescence in the ubiquitin channel nor ubiquitin-positive puncta formation, indicating that the observed effect on the ubiquitin signal was associated to DOPAL.

**Fig. 5:**
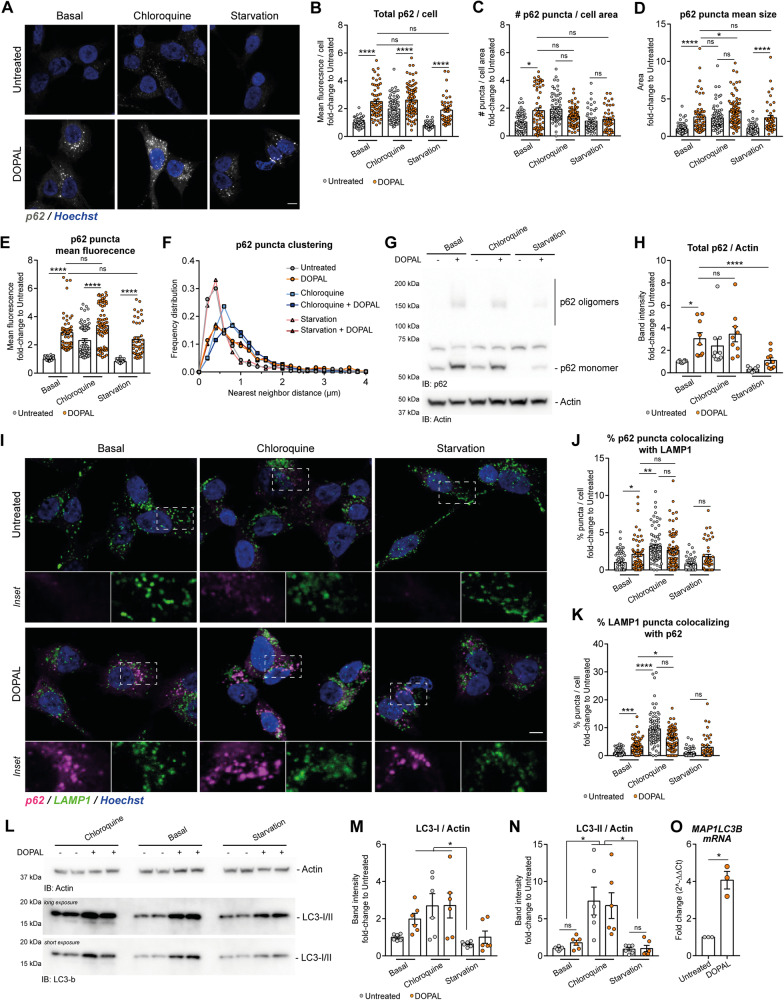
DOPAL affects p62 routing along the autophagic pathway. **A** Immunofluorescence of p62 cytosolic distribution (gray) in untreated and 100 µM DOPAL-treated cells, both in basal conditions and after 50 µM chloroquine and serum starvation treatments for 18 h. The nuclei are stained with Hoechst (blue). Scale bar: 5 µm. Relative quantification of **B** total p62 (mean fluorescence intensity)/cell, **C** number of puncta/cell area, **D** mean puncta size, **E** mean puncta fluorescence, and **F** p62 puncta clustering expressed as frequency distribution of the Nnd (μm) among puncta. Data are pooled together from three independent experiments normalized to each untreated sample, and analyzed by Kruskall–Wallis test with Dunn’s multiple comparison test (*P < 0.05, ****P < 0.0001). **G** Western blot of p62 in BE(2)-M17 cells treated in the same conditions and **H** relative quantification after normalization to Actin used as loading control. Data are pooled together from five independent experiments normalized to each untreated sample, and analyzed by the Kruskall–Wallis test with Dunn’s multiple comparison test (*P < 0.05, ****P < 0.0001). **I** Immunofluorescence of p62-positive puncta (magenta) and LAMP1-positive structures (green) in untreated and 100 µM DOPAL-treated cells, both in basal conditions and after 50 µM chloroquine and serum starvation treatments for 18 h. The nuclei are stained with Hoechst (blue). Scale bar: 5 µm. In the insets, the overlay images are split in the p62 and LAMP1 channels. **J**, **K** Quantification of the percentage of p62-positive puncta co-localizing with LAMP1-positive structures in each cell and vice versa. Data are pooled together from three independent experiments normalized to each untreated sample, and analyzed by Kruskall–Wallis test with Dunn’s multiple comparison test (*P < 0.05, **P < 0.01, ***P < 0.001, ****P < 0.0001). **L** Western blot of LC3b in BE(2)-M17 cells treated in the same conditions and **M**, **N** relative quantification of LC3-I and LC3-II, respectively, after normalization to Actin used as loading control. Data are pooled together from three independent experiments normalized to each untreated sample, and analyzed by Kruskall–Wallis test with Dunn’s multiple comparison test (*P < 0.05). **O** Real-time PCR of *MAP1LC3B* mRNA levels in untreated and 100 µM DOPAL for 18 h. Data are pooled together from three independent experiments normalized to each untreated sample, and analyzed by one-sample *t* test (*P < 0.05).

We then used p62 itself to study its degradation by autophagy, also as readout of the functionality of this degradative pathway. We analyzed more in detail the variation in p62 levels and clustering in cytosolic puncta induced by DOPAL in basal conditions, upon autophagy blockage by chloroquine and autophagy activation by serum starvation (Fig. 5A). By immunofluorescence analysis, DOPAL buildup resulted in a significant increase in total p62 levels, number of p62 puncta per cell area, p62 puncta dimension and mean fluorescence intensity, with the same trend under chloroquine treatments (Fig. 5B–E). Of note, the analysis of p62 clustering measured as frequency distribution of the nearest neighbor distance (Nnd) among puncta, revealed consistently scattered structures in chloroquine-treated and all DOPAL-treated cells, while serum starvation displayed the tendency to maintain p62 puncta closer to one another like in untreated cells (Fig. 5F). Similarly, the immunoblot analysis on lysates derived from cells treated in the same conditions, revealed a significant accumulation of p62 in both DOPAL and chloroquine-treated cells (Fig. 5G, H). While serum starvation promoted p62 clearance in both experiments, in the presence of DOPAL a fraction of p62 monomer, oligomers and clusters was still present, appearing more resistant to degradation. Hence, we studied the co-localization with the lysosomal marker LAMP1 (lysosomal-associated membrane protein 1) upon modulation of the autophagic flux under the same conditions (Fig. 5I). Consistently, DOPAL treatment led to a significant accumulation of p62 within lysosomal-like structures, as shown by the quantification of the percentage of p62 puncta co-localizing with LAMP1 puncta, and vice versa, while autophagy inhibition by chloroquine displayed a significantly higher effect (Fig. 5J, K).

Based on the observed p62 oligomers and clusters still present under serum starvation and DOPAL treatment, we reasoned that DOPAL-modified p62 could be not only less degradable but also less functional, thus hindering the autophagic machinery. To explore this possibility, we measured the levels of the autophagic marker and substrate LC3b, which should accumulate if autophagy is compromised. Similar to p62, also LC3b levels increased in the presence of DOPAL, and starvation only partially reduced LC3b-I levels compare to untreated cells, corroborating the notion that the autophagic process is compromised per se (Fig. 5L–N). Further supporting this scenario, *MAP1LC3B* mRNA levels were significantly upregulated upon DOPAL treatment (Fig. 5O), possibly indicating that the cell is attempting to boost autophagy to compensate for reduced protein clearance.

**Fig. 6:**
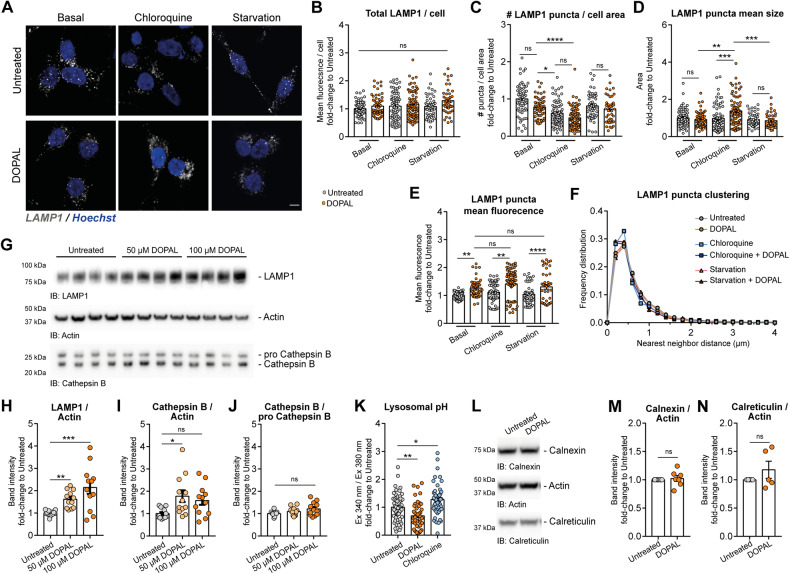
DOPAL effect on lysosomes. **A** Immunofluorescence of LAMP1-positive endolysosomes (gray) in untreated and 100 µM DOPAL-treated cells, both in basal conditions and after 50 µM chloroquine and serum starvation treatments for 18 h. The nuclei are stained with Hoechst (blue). Scale bar: 5 µm. Relative quantification of **B** total LAMP1 (mean fluorescence intensity)/cell, **C** number of puncta/cell area, **D** mean puncta size, **E** mean puncta fluorescence, and **F** LAMP1 puncta clustering expressed as the frequency distribution of the Nnd (μm) among puncta. Data are pooled together from three independent experiments normalized to each untreated sample, and analyzed by Kruskall–Wallis test with Dunn’s multiple comparison test (*P < 0.05, **P < 0.01, ****P < 0.0001). **G** Western blot of p62 in BE(2)-M17 cells treated with 0–50–100 µM DOPAL for 18 h. Relative quantification of **H** LAMP1 and **I** active Cathepsin B after normalization to Actin used as loading control, and **J** ratio between active and pro-Cathepsin B. Data are pooled together from three independent experiments normalized to each untreated sample, and analyzed by Kruskall–Wallis test with Dunn’s multiple comparison test (*P < 0.05, **P < 0.01, ***P < 0.001). **K** Measurement of lysosomal pH using the ratiometric probe Lysosensor in BE(2)-M17 cells treated with 100 µM DOPAL or 50 µM chloroquine for 18 h, and the untreated control. The values represent the ratio of fluorescence emission (490 nm) after excitation at 340 nm and 380 nm for each sample. Data are pooled together from three independent experiments normalized to each untreated sample, and analyzed by Kruskall–Wallis test with Dunn’s multiple comparison test (*P < 0.05, **P < 0.01). **L** Immunoblot of Calnexin and Calreticulin in untreated and 100 µM DOPAL-treated cells and **M**, **N** relative quantification after normalization to Actin used as loading control. Data are pooled together from three independent experiments normalized to each untreated sample and analyzed by one-sample *t* test (ns *P* > 0.05).

To understand whether DOPAL-induced autophagy dysfunction impacted lysosomal functionality, we investigated any alteration at the lysosomal level due to the DOPAL-induced clogging of p62 oligomers in this organelle. To this aim, we performed a similar analysis on LAMP1-positive puncta as we did for p62, evaluating total LAMP1 fluorescence/cell, the number of LAMP1 puncta/cell area, the mean puncta size and fluorescence, and the LAMP1 puncta clustering (analyzed as the frequency distribution of the nearest neighbor distance among puncta) in the cytoplasm (Fig. 6A–F). DOPAL did not appear to promote significant variations for all parameters, except for a systematic increase in mean puncta fluorescence in basal, chloroquine-treated and serum-starved cells (Fig. 6E). The higher levels of LAMP1 were also confirmed by western blot, in a dose-dependent trend (Fig. 6G, H). In the same experiment, the activation of Cathepsin B by the cleavage of the pro-Cathepsin B by lysosomal proteases [40], used as indirect readout of lysosomal functionality, did not reveal any significant alteration (Fig. 6I, J), indicating that there are not overt abnormalities. On the contrary, the analysis of lysosomal pH using the ratiometric probe Lysosensor, revealed a significant increase in the acidification of lysosomes in DOPAL-treated cells, while the pH value increased with the chloroquine treatment used as positive control in the assay (Fig. 6K). This could be possibly ascribed to altered lysosomal physiology in the attempt at degrading DOPAL-p62 oligomers. Taken together, the overall effect of DOPAL buildup on p62 role and trafficking along the autophagic pathway appeared to compromise the initial phases of the autophagic flux at the level of the autophagosomes, with minor consequences at the lysosomal compartments in the time frame of our experimental setup.

Finally, to check whether DOPAL impact on cellular proteostasis could also involve the endoplasmic reticulum (ER) leading to ER stress and the activation of the unfolded protein response (UPR), we looked at the total level of calnexin and calreticulin in the cell lysate (Fig. 6L). As no significant increase in the ER markers was detected in DOPAL-treated cells (Fig. 6M, N), we concluded that DOPAL mainly affects the functionality of protein degradative pathways.

### αSynuclein buildup enhances DOPAL-induced p62 oligomerization

**Fig. 7:**
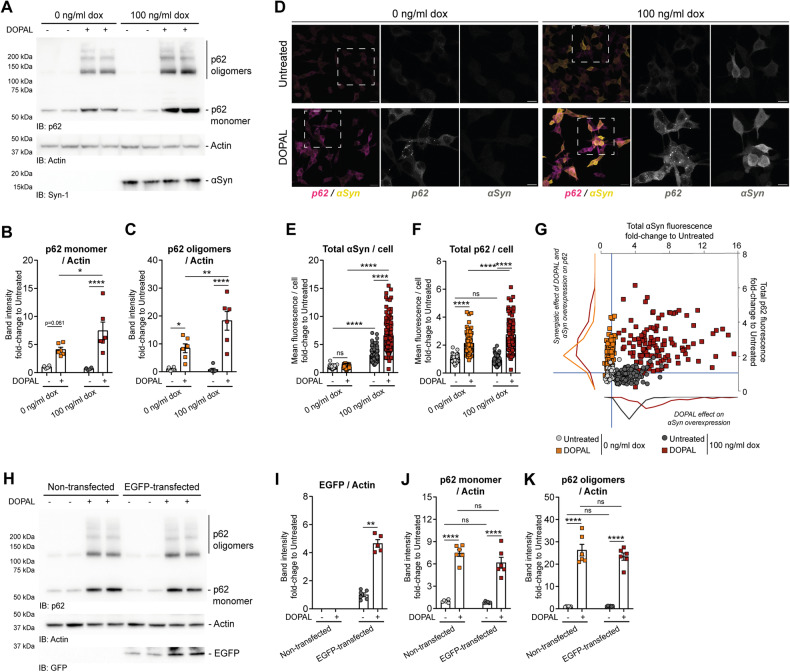
αSynuclein buildup enhances DOPAL-induced p62 oligomerization. **A** Immunoblot of stable BE(2)-M17 cells with inducible dox-dependent αSyn overexpression, both in untreated and 100 µM DOPAL-treated for 18 h conditions. Relative quantification of **B** p62 monomer and **C** p62 oligomers after normalization to Actin used as loading control. Data are pooled together from four independent experiments normalized to each untreated sample, and analyzed by two-way ANOVA with Tukey’s multiple comparison test (*P < 0.05, **P < 0.01, ****P < 0.0001). **D** Immunofluorescence of p62 (magenta) and αSyn (green) in the same cellular model. Scale bar: 20 µm and 5 µm in the inset. Relative quantification of **E** αSyn mean fluorescence/cell and **F** p62 mean fluorescence/cell. Data are pooled together from four independent experiments normalized to each untreated sample, and analyzed by two-way ANOVA with Sidak’s multiple comparison test (****P < 0.0001). **G** Correlation of αSyn and p62 mean fluorescence in each cell. The blue lines are centered on the reference level (normalization to 1) for each axis. On the bottom part, frequency distribution of αSyn fluorescence in overexpressing cells by 100 ng/ml dox, showing the effect of DOPAL on αSyn accumulation. On the left side of the graph, frequency distribution of p62 fluorescence in DOPAL-treated cells, showing the synergistic effect of DOPAL and αSyn overexpression on p62 buildup. **H** Immunoblot of non-transfected and EGFP-transfected BE(2)-M17 cells, both in untreated and 100 µM DOPAL-treated for 18 h conditions. Relative quantification of **I** EGFP, **J** p62 monomer, and **K** p62 oligomers after normalization to Actin used as loading control. Data are pooled together from three independent experiments normalized to each non-transfected untreated sample, and analyzed by two-way ANOVA with Tukey’s multiple comparison test (**P < 0.001, ****P < 0.0001).

Our previous work suggests that the detrimental effect of DOPAL-induced p62 accumulation in the soma of neurons is mitigated by the absence of αSyn expression [14]. Here, we sought to further explore the synergistic interplay between DOPAL and DOPAL-induced αSyn buildup on p62-altered proteostasis. To this aim, we analyzed the variations in p62 monomeric and oligomeric species upon DOPAL treatment in a stable BE(2)-M17 cell line overexpressing αSyn under a dox-inducible promoter (Fig. 7A). In this paradigm, we detected a cumulative effect of DOPAL and αSyn overexpression induced by 100 ng/ml dox, leading to a significant increase in both p62 monomer and oligomers (Fig. 7B, C). Consistent results were obtained by the immunofluorescence study of αSyn and p62 in the same experimental setup (Fig. 7D). Together with a significant increase in αSyn fluorescence in overexpressing DOPAL-treated cells (Fig. 7E), the DOPAL effect on p62 buildup was significantly aggravated in dox-induced cells (Fig. 7F). The interplay among DOPAL, αSyn and p62 is clearly visualized by the αSyn/p62 fluorescence correlation in which each cell is reported under the different conditions (Fig. 7G). The frequency distribution of αSyn fluorescence in dox-induced cell (in the bottom part) highlighted the effect of DOPAL treatment in accumulating αSyn in the overexpressing system, while DOPAL-induced p62 buildup did not affect αSyn endogenous levels. Nevertheless, while αSyn overexpression per se did not cause any variation on p62 levels in basal condition, DOPAL treatment and DOPAL-dependent αSyn accumulation had a synergistic effect on p62 increase (frequency distribution of p62 fluorescence on the left side of the graph). Importantly, we then assessed whether this effect was due to the overexpression of a protein per se, rather than being specific to αSyn. Hence, we repeated the experiment inducing the overexpression of EGFP (Fig. 7H), which is reported to be primarily degraded by the proteasome [41]. While DOPAL treatment induced a 5-fold increase in EGFP levels (Fig. 7I), we did not observe a significant difference on p62 monomer nor oligomers between DOPAL-treated non-transfected and EGFP-overexpressing cells (Fig. 7J, K).

Altogether, these data further support the neurotoxic αSyn and DOPAL interplay, which triggers detrimental downstream effects, including p62 oligomerization and impaired autophagy degradation.

## Discussion

**Fig. 8:**
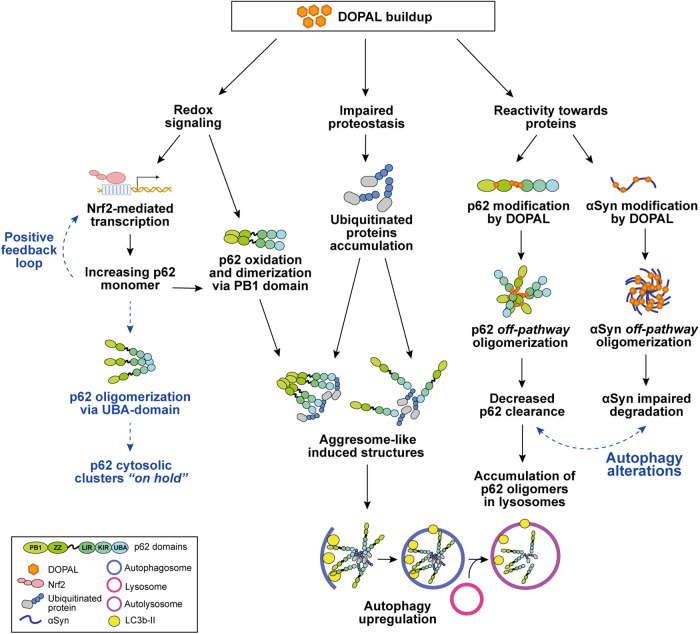
Schematic representation of the working hypothesis. Multiple molecular mechanisms triggered by DOPAL cellular buildup, which may account for p62 oligomerization and clustering, and possible downstream effects (in blue).

In this study, we demonstrated that the pathological accumulation of the dopamine catabolite DOPAL in cells, associated to the dopaminergic dysfunction in PD, displays a distinct effect on the scaffold protein p62, leading to an altered proteostasis at different levels. Both in vivo and cellular models of DOPAL buildup showed a significant increase in p62 amount, associated to protein oligomerization and clustering in cytosolic puncta. Based on the present findings, we hypothesized a convergence of multiple molecular mechanisms that generate the observed p62 phenotype induced by DOPAL, as we summarized in Fig. 8 and discuss below.

Starting from the observation that DOPAL induces p62 cytoplasmic clusters buildup, we investigated the possible mechanisms that may lead to this phenotype. p62 bodies have been previously described in several studies as a result of increased protein synthesis, autophagy alterations or in response to different cellular stressors [31, 42–46]. p62 bodies mostly co-localize with ubiquitin as p62 UBA domain recognizes poly-ubiquitinated proteins, with at least three ubiquitin moieties per chain, to be then delivered to degradative pathways [46]. Therefore, p62 can either shuttle short-lived single-molecule proteins to the proteasome, interacting with the 26 S subunit via the N-terminal PB1 domain; alternatively, it associates to K63-poly-ubiquitinated protein aggregates that require autophagic clearance, by binding via the LIR domain to LC3b-II on the autophagosomal membrane formation [39]. Moreover, p62 itself can oligomerize via the PB1 domain and act as nucleating seed to recruit ubiquitinated proteins and organelles in a structure also known as sequestosome or aggresome-like induced structure (ALIS), which will be subsequentially engulfed by the autophagosomes [39, 42]. In this scenario, p62 represents a sensor for oxidative stress in the cell due to a pair of cysteine residues at the N-terminus (Cys105 and Cys113) that are oxidation-sensitive [43]. The formation of disulfide bonds between oxidized cysteines of p62 monomers promotes PB1-ZZ homodimerization and accelerates p62 oligomerization to induce pro-survival autophagy activation under stress conditions.

In our experiments, we detected DOPAL-induced p62 clusters co-localizing with ubiquitin, and DOPAL-associated redox chemistry might contribute to PB1-mediated p62 oligomerization. In addition, an alternative p62 homodimerization mechanism has been described, which involves non-covalent interactions at the level of the UBA domain, generating cytosolic membrane-free p62 gels with liquid-liquid phase separation [44]. In the latter complex, p62 is hindered in both its interaction with ubiquitinated proteins and in its function in autophagy. This “holding state” is reversible, as a series of phosphorylation at the C-terminal region i.e., pSer403 and pSer407 by Unc-51 like autophagy activating kinase 1 (ULK1), weakens the UBA interactions, thus promoting the binding to ubiquitinated proteins and the autophagic flux [44]. On these premises, it will be interesting to further investigate whether at least a fraction of p62 bodies generated by DOPAL undergoes the same fate, thus accumulating non-functional p62 in the cytosol.

The second piece of evidence, which corroborates and expands the immunofluorescence data, is the detection of increased p62 protein levels by western blot in DOPAL-treated cells, both monomeric and SDS-resistant oligomers, with a specific effect as compared to other cytosolic proteins. Based on our data, the higher levels of monomeric p62 can be ascribed to the activation of the Nrf2 pathway, that we found to be significantly elevated with DOPAL treatment. In this frame, we speculate that the sustained p62 expression could be self-induced in a positive feedback loop. This would lead to p62 association to Keap1 through the KIR domain and induce the disassembly of the of Nrf2-Keap1 complex, thus allowing Nrf2 translocation into the nucleus and binding to the ARE sequences upstream *SQSTM1* [31]. Although Nrf2-mediated transcription of antioxidant and anti-inflammatory proteins under stress conditions has a protective role, the persistent activation of the Nrf2 pathway has been positively correlated with PD pathology in experimental models and PD duration (years from clinical diagnosis) in patients’ studies [47, 48]. On the other hand, we cannot exclude the activation of the CLEAR (Coordinated Lysosomal Expression and Regulation) gene network by its master gene transcription factor EB (TFEB), leading to increased p62 expression in DOPAL-treated cells [49, 50]. This would fit with the hypothesis of an upregulation of autophagy in the attempt to degrade accumulating proteins due to DOPAL-induced proteotoxicity, as we discuss further below and which will be interesting to unravel in the future.

Nevertheless, our imaging analysis with the 8xARE-GFP reporter demonstrated that the activation of the Nrf2 pathway non-necessarily correlated with the formation of p62 clusters, which were specifically detected in DOPAL-treated cells as compared to DMF-exposed cells. Similar multimeric p62 species have been detected in the brain of mice with impaired autophagy [45], or resolved by SDS-PAGE in non-reducing conditions from cell lysates exposed to inhibitors of the autophagic pathway (chloroquine and bafilomycin) and oxidant agents (H_2_O_2_ and PR-619) [43]. However, in our hands, such treatments did not generate stable p62 multimers to the level of DOPAL, which was much more effective. Moreover, DOPAL-induced p62 oligomers did not present the filamentous structure previously described for p62 by TEM studies [46, 51]. Hence, we hypothesized a DOPAL-induced p62 *off-pathway* oligomerization, as it was previously described for DOPAL-modified αSyn oligomers which do not present a specific folding or structural pattern [13].

Interestingly, both the protein pull-down with the APBA resin and the detection of the nIRF signal indicated a direct DOPAL modification on p62, and our MS/MS analysis identified few residues on p62 sequence that were modified by DOPAL. Of note, four residues were DOPAL-modified via the catechol moiety: Cys113, which is within the predicted unfolded loop between the PB1 and the ZZ domain; Lys281, Cys289, Lys295 which map in the unfolded region in the central portion of the protein, as highlighted by the model predicted by AlphaFold (AF-Q13501-F1). The two lysine residues (Lys281 and Lys295) were also modified by Schiff base. Even Lys187 and Lys189, which were modified by DOPAL in a loop-linked peptide, are present in an unfolded region of the protein. Notably, the loop-linked Lys100 and Lys103 are located in a region between PB1 and UBA domain. Unfortunately, intermolecular cross-links were not detected by mass spectrometry, likely due to (i) the difficulty to ionize the resulting high- molecular-weight peptides; (ii) the low abundance of each type of intermolecular cross-linked peptides resulting from the several ways of DOPAL reaction occurring. In any case, the detection of loop-linked peptides in the monomeric form of p62 suggests which regions of the protein are more prone to DOPAL-induced oligomerization.

Until recently, only the structures of the distinct p62 domains were available and either obtained by EM (aa 1–122, corresponding to the PB1 domain), X-ray (aa 126–180, corresponding to the ZZ domain) or NMR (aa 387–436, corresponding to the UBA domain) (source: rcsb.org). Hence, AlphaFold prediction allows to expand the spatial visualization to natively unstructured proteins or domains, and to residues that appear quite exposed to the surrounding environment and accessible to modification. Moreover, DOPAL promoted the oxidation to sulfoxide of several methionine, which could modulate the aggregation propensity of p62 as previously reported for αSyn [52]. In this frame, catechol oxidation and ROS generation is quite relevant as nigrostriatal dopaminergic neurons are particularly susceptible to oxidative stress, due to compromised antioxidant defenses (relative low levels of GSH) and reduced expression of key enzymes like superoxide dismutase, glutathione reductase, and glutathione peroxidase [53]. It is also worth pointing out that the catechol moiety is present in both DOPAL and dopamine molecular structures and a similar covalent reaction could be responsible for p62 oligomeric species that we detected by western blot in dopamine-treated cells, although DOPAL displays a significantly higher reactivity towards p62 as pointed out by the different fold change. Also, the antioxidant environment generated by NAC, Asc, and GSH could attenuate the dopamine effect on p62 while they did not prevent DOPAL-induced p62 oligomerization. This is potentially due to the protein cross-linking generated by Schiff base reactions via the aldehyde moiety, which is only present in DOPAL and not in dopamine.

With regard to DOPAL modification sites, we identified Cys113 which is directly implicated in p62 homodimerization via PB1 domain upon oxidation and disulfide bond formation [43]. Hence, we reasoned that DOPAL could accelerate and stabilize p62 oligomerization acting as cross-linker. In addition, the other three modified residues appear quite close to important phosphorylation sites that regulate p62 function and localization [15]. For instance, Lys295 is in close proximity to Ser294, which is phosphorylated by AMP-activated protein kinase (AMPK) to promote autophagy and mitophagy. Thus, any steric hindrance around these modification sites could interfere with or modulate important signaling pathways.

We then explored the physiological implications of DOPAL-dependent p62 oligomers buildup with focus on p62 routing along the autophagic pathway. In our experiments, we detected DOPAL-induced p62 clusters co-localizing with ubiquitin, which possibly suggests the attempt to activate the autophagic flux to promote misfolded protein clearance, consistent with the significant accumulation of ubiquitinated proteins measured in the total cell lysates and the upregulation of LC3b. In addition, we observed increasing p62 puncta also co-localizing with LAMP1-positive structures upon DOPAL treatment, depicting a scenario similar to the impaired autophagic flux induced by chloroquine. Of note, while serum starvation efficiently degraded the majority of p62 leaving less protein available for modification, in the presence of DOPAL a residual fraction of oligomeric species and clusters appeared resistant to degradation. Hence, we hypothesized that DOPAL causes a certain degree of loss-of-function of p62 which, over time, could affect the functionality of protein quality control and autophagic clearance capacity of the cell. In our experimental conditions, DOPAL impact on the degradative machinery appeared to mainly involve the first steps of the autophagic flux, while the consequences at the lysosomal level are more difficult to interpret and will need further investigations.

Overall, DOPAL-induced p62 impaired proteostasis could have detrimental outcomes on dopaminergic neuron survival. PD-associated mitochondrial damage and oxidative stress are known to activate parkin-mediated mitophagy, but a dysregulation of parkin–p62 axis could have important consequences on mitochondrial turnover [21]. Similarly, while interacting with ubiquitinated misfolded proteins and aggregates, DOPAL-induced oligomeric p62 could prevent their degradation and promote their deposition in cytoplasmic inclusions [22, 23, 54, 55]. In addition, oxidative stress-induced S-nitrosylation of p62, which is increased in human LBs, has been demonstrated to block the autophagic flux and to promote αSyn extracellular release [56]. Since we recently demonstrated that DOPAL buildup promotes αSyn secretion via the exosomal pathway [14], it may be speculated that DOPAL-modified p62 may contribute to this spreading mechanism.

Finally, we demonstrated a specific pathological interplay among DOPAL, αSyn, and p62, in which DOPAL buildup and DOPAL-induced αSyn accumulation act in synergy to exacerbate p62 oligomerization and clustering. αSyn is highly concentrated at synaptic level, where also DOPAL is generated, and based on the high percentage of lysines and the intrinsically unfolded nature, αSyn represents a preferential target for DOPAL reactivity [57]. DOPAL-αSyn oligomerization and consequent synaptic impaired proteostasis, possibly recruits p62 and the autophagosomal machinery in the neuronal projections, to promote the clearance of neurotoxic aggregates via autophagy. In this scenario, the local accumulation of p62 facilitates its modification by DOPAL, triggering p62 *off-pathway* oligomerization and the molecular mechanisms we described in this study. In addition, p62 has been reported to have a fast turnover in the range of 2–6 h [42, 43], which continuously provides new substrate for modification.

Interestingly, p62 displays similar features as αSyn, among which the intrinsic tendency to homo-oligomerization and the presence of modification sites within unstructured regions of the protein. In the future, it will be interesting to screen the neuronal proteome to identify other proteins with natively unfolded domains which are potential targets of DOPAL reactivity. This will allow to further assess the impact of natively unfolded domains altered proteostasis on dopaminergic neurons, thus paving the way to a better understanding of PD pathology.

### Supplementary information

Supplementary Information

## Data Availability

Most data generated or analyzed are included in the article. Additional datasets and analyses, as well as relevant information reported in this paper, could be shared by the corresponding author upon request.

## Materials availability

All unique reagents (e.g., plasmids) generated in this study are available from the corresponding author without restriction.
