# Supplementary material for: Sequestosome-1 (SQSTM1/p62) as a target in dopamine catabolite-mediated cellular dyshomeostasis
Source: Cell Death Dis. 2024 Jun 18;15(6):424. doi: 10.1038/s41419-024-06763-x (PMC11189528; doi:10.1038/s41419-024-06763-x)
Supplement: Supplementary file 1 — Supplementary Information [file 41419_2024_6763_MOESM1_ESM.docx]

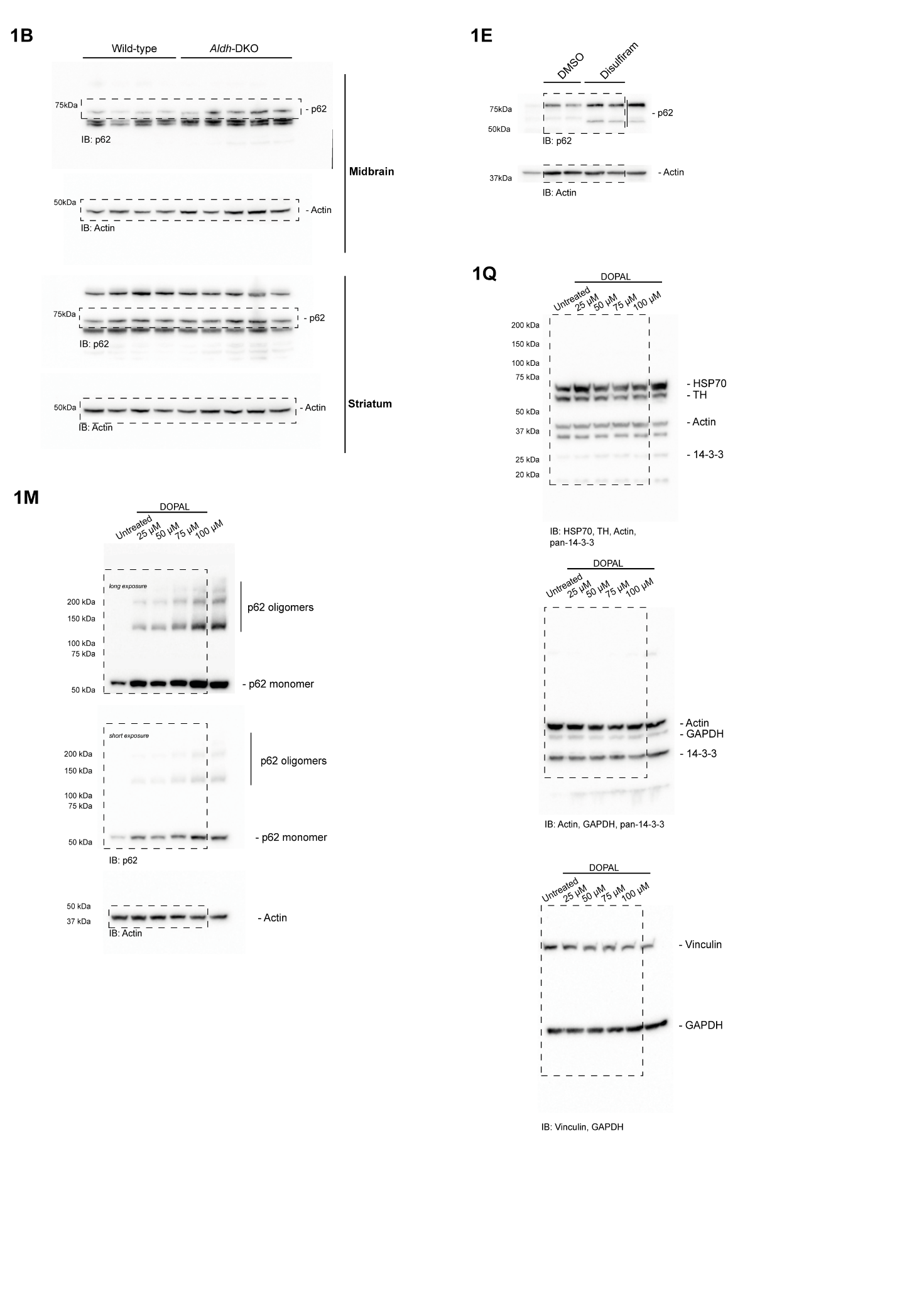


Full-length uncropped western blot from **Figure 1.**


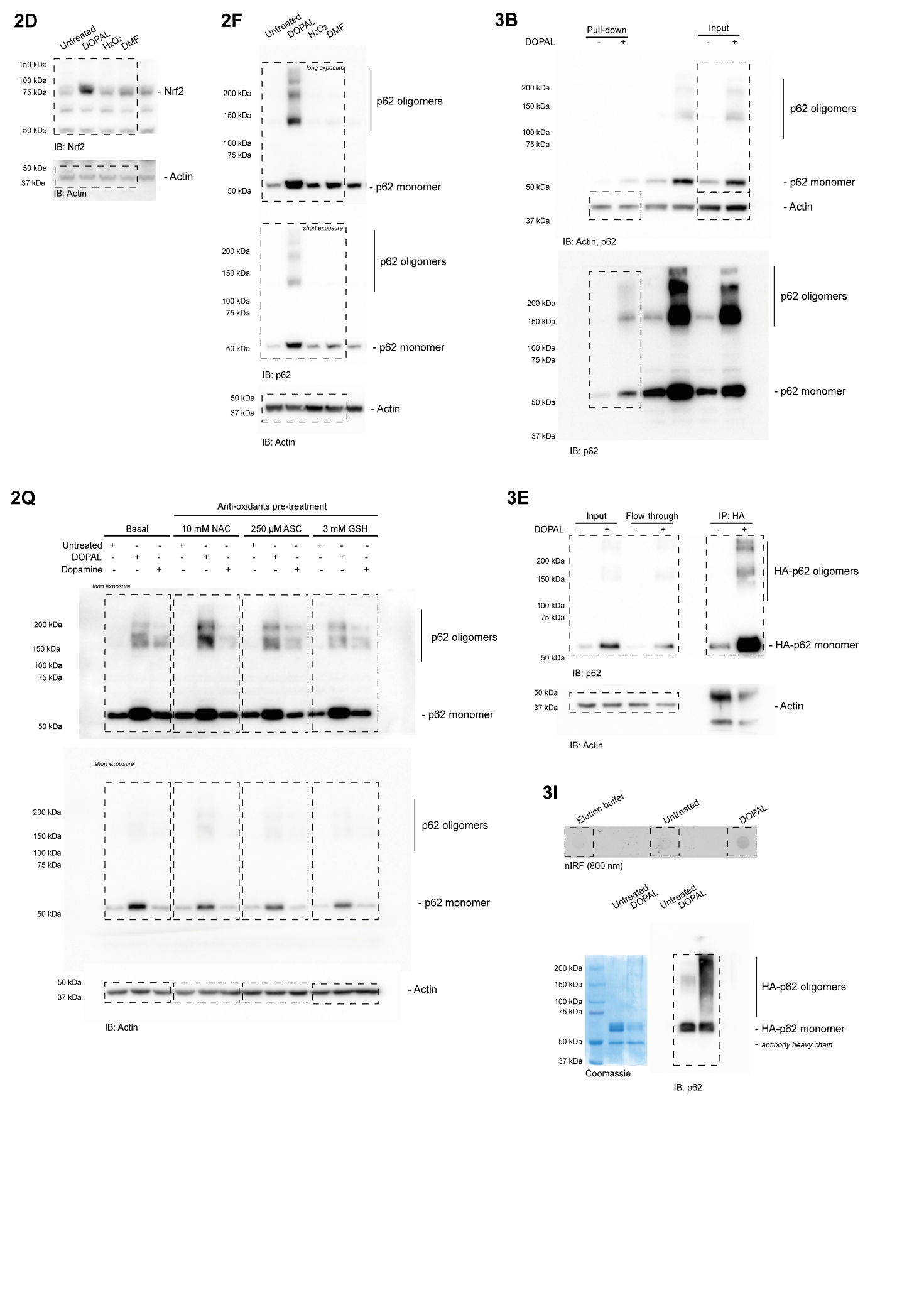
Full-length uncropped western blot from **Figures 2-3.**


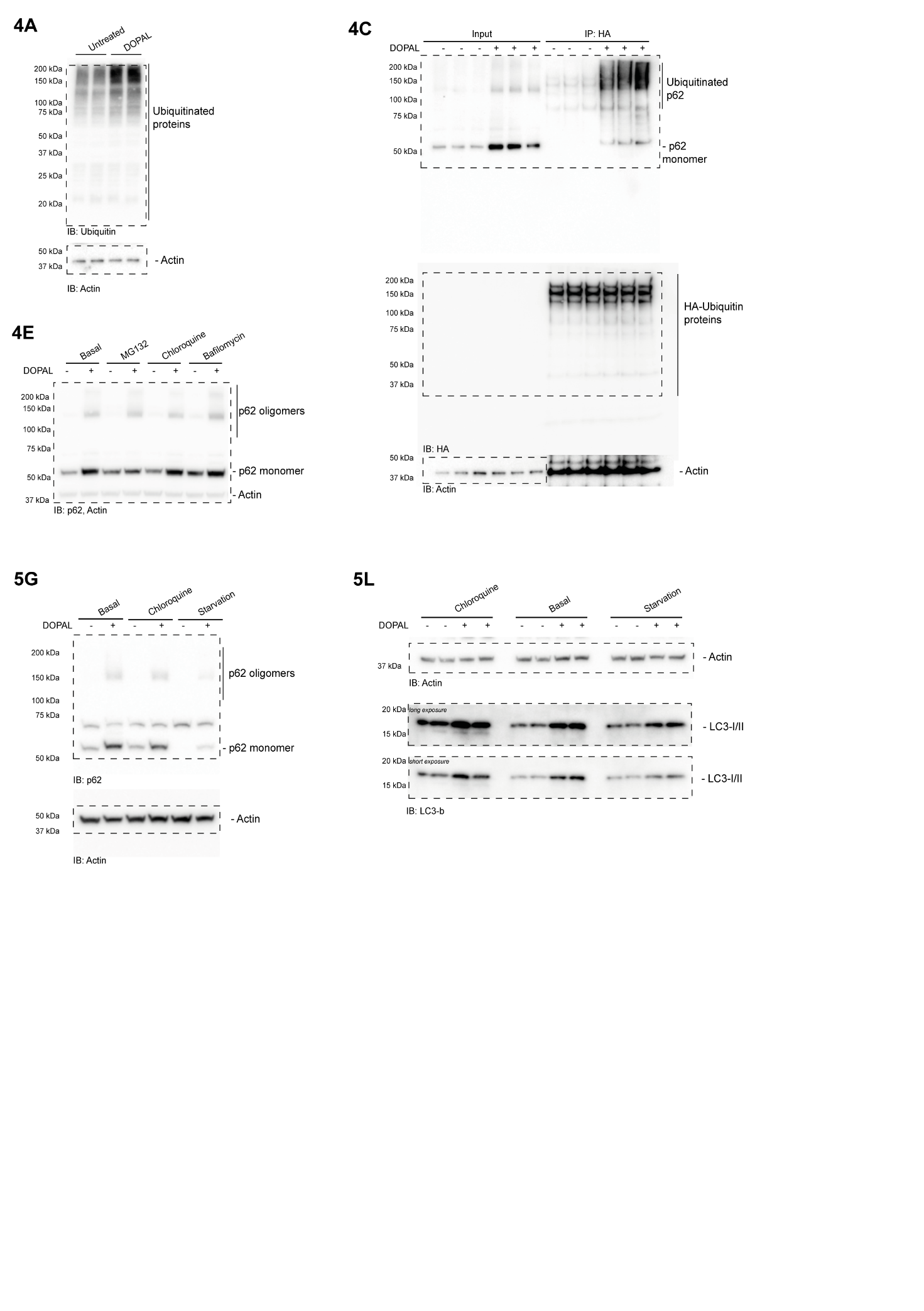
Full-length uncropped western blot from **Figures 4-5.**


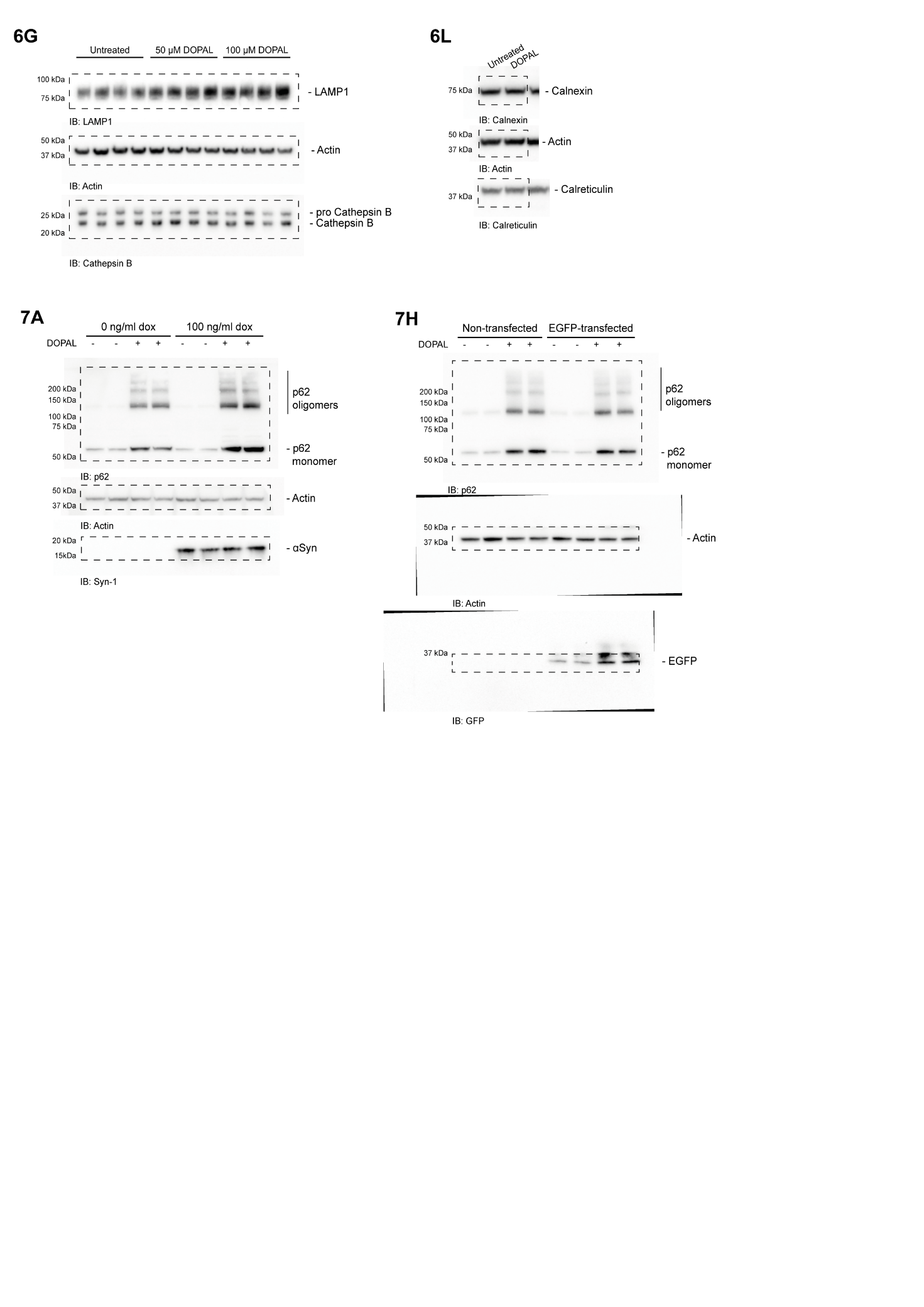
Full-length uncropped western blot from **Figures 6-7.**
